# Supplementary figures and images for: Deciphering the mechanism of anhydrobiosis in the entomopathogenic nematode Heterorhabditis indica through comparative transcriptomics
Source: PLoS One. 2022 Oct 27;17(10):e0275342. doi: 10.1371/journal.pone.0275342 (PMC9612587; doi:10.1371/journal.pone.0275342)

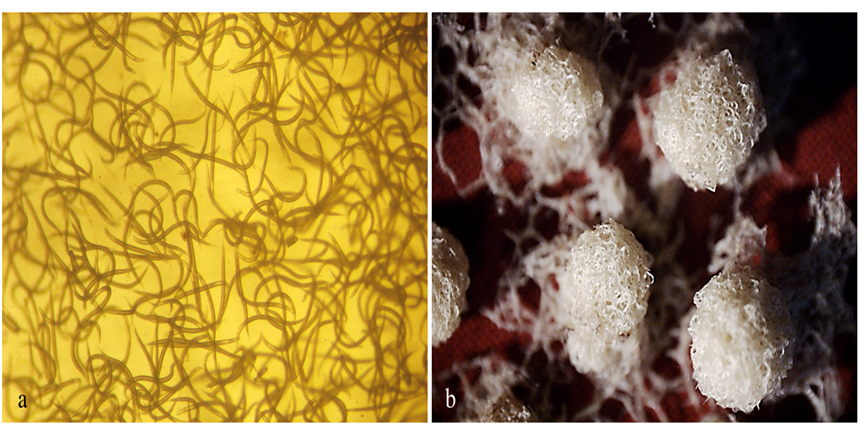

Supplement: S1 Plate — (TIF) [file pone.0275342.s001.tif]

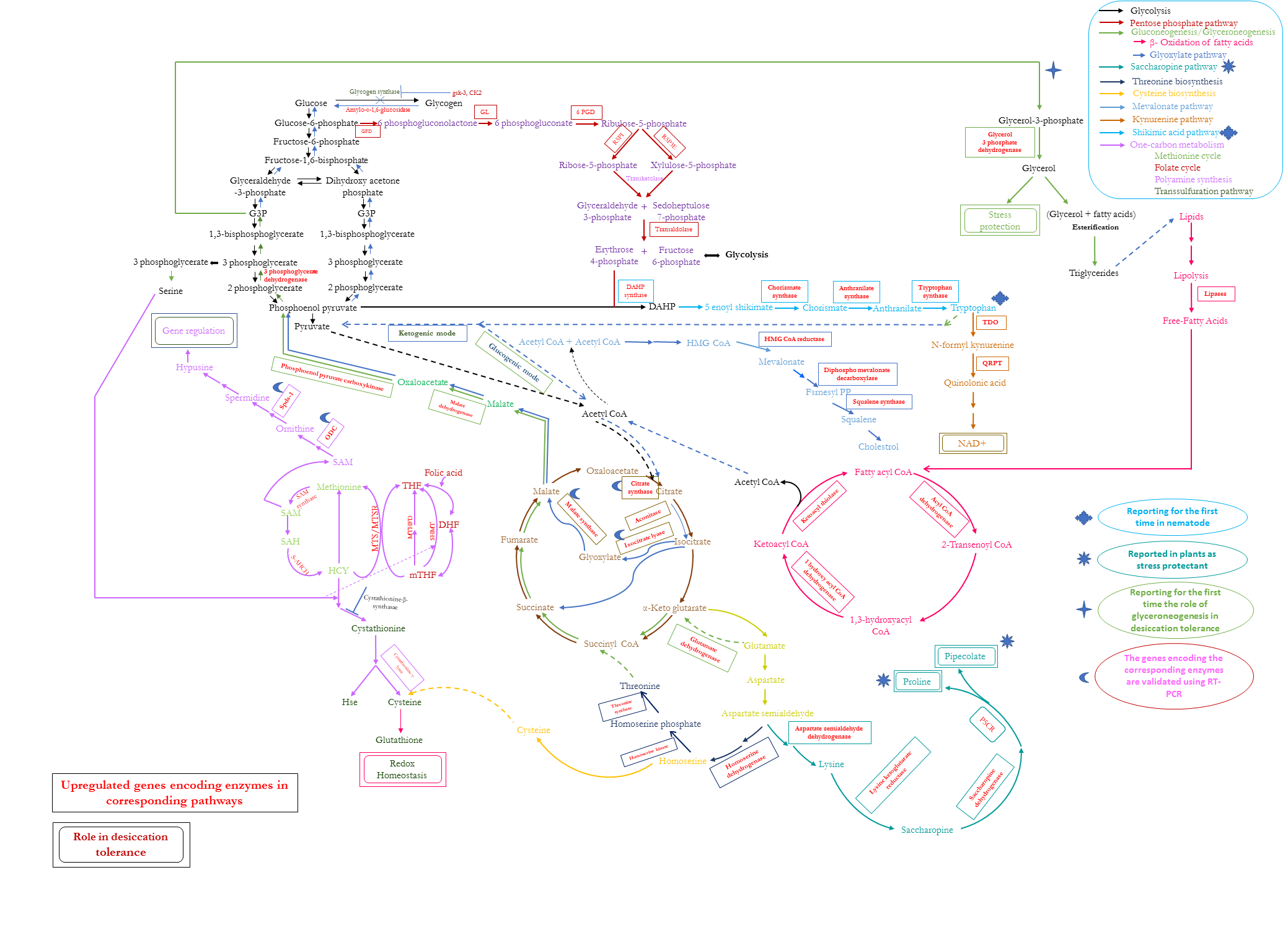

Supplement: S2 Plate — Heterorhabditis indica infective stage juveniles (a) unstressed (b) anhydrobiotic (x20). (TIF) [file pone.0275342.s002.tif]

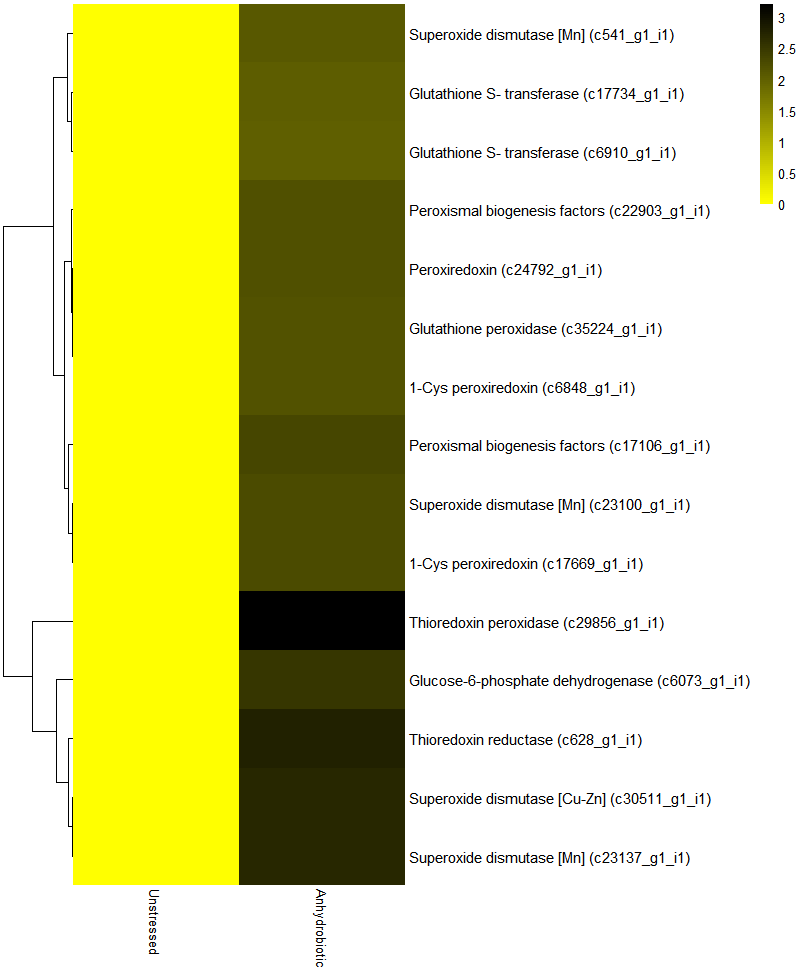

Supplement: S1 Fig — (TIF) [file pone.0275342.s007.tif]

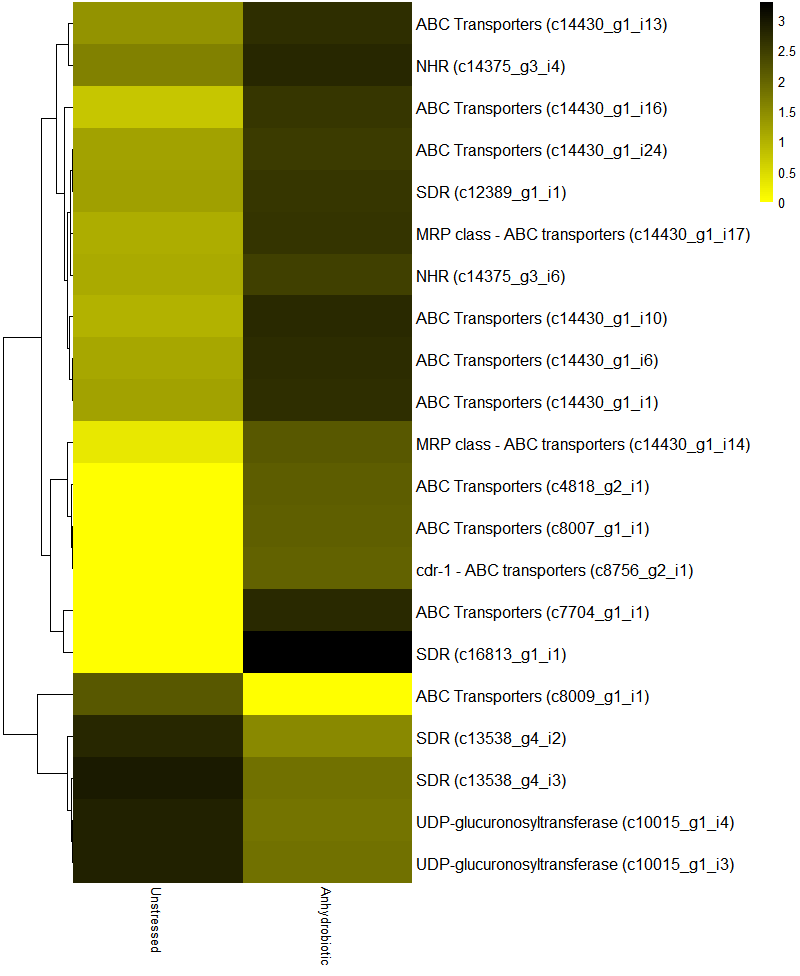

Supplement: S2 Fig — (TIF) [file pone.0275342.s008.tif]

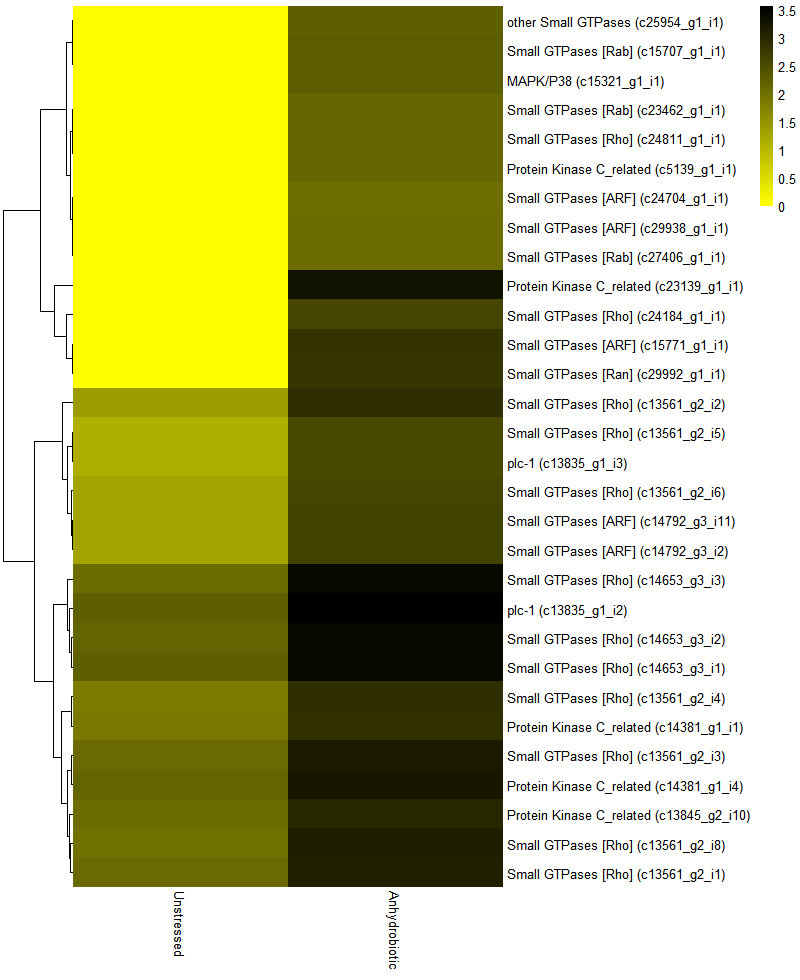

Supplement: S3 Fig — (TIF) [file pone.0275342.s009.tif]

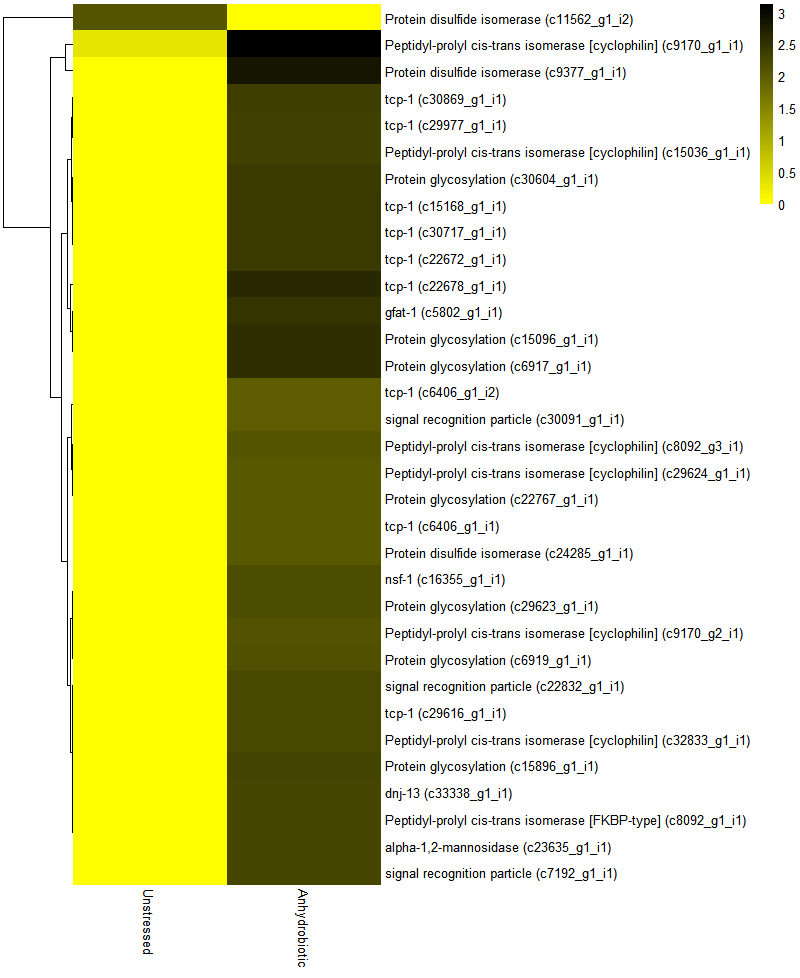

Supplement: S4 Fig — (TIF) [file pone.0275342.s010.tif]

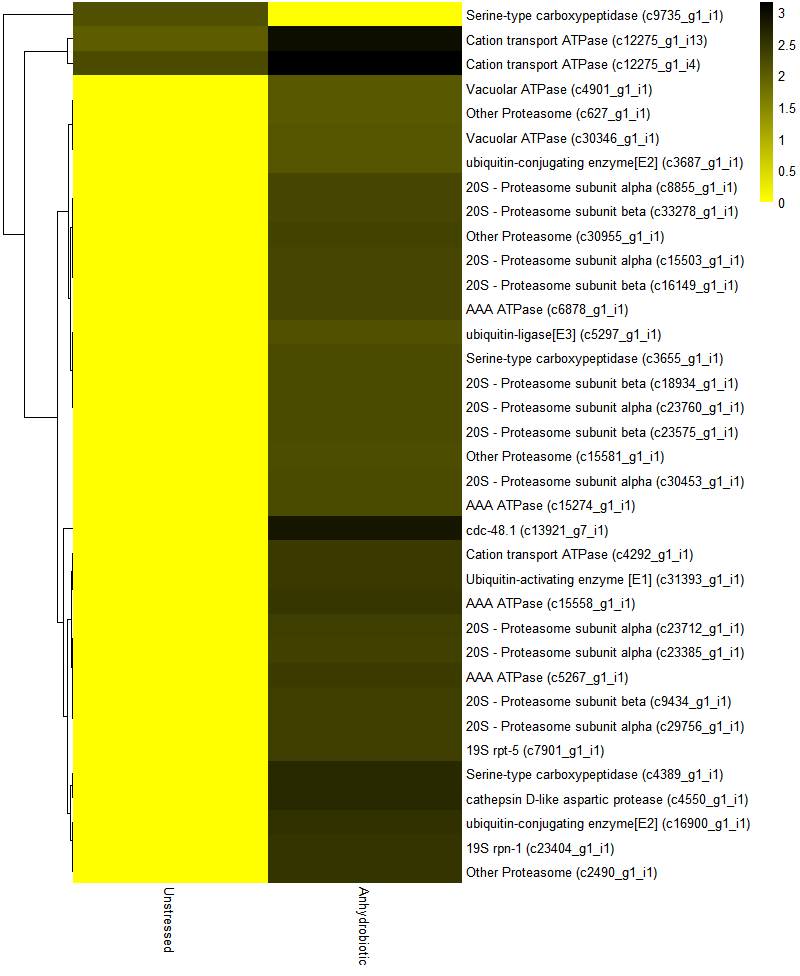

Supplement: S5 Fig — (TIF) [file pone.0275342.s011.tif]

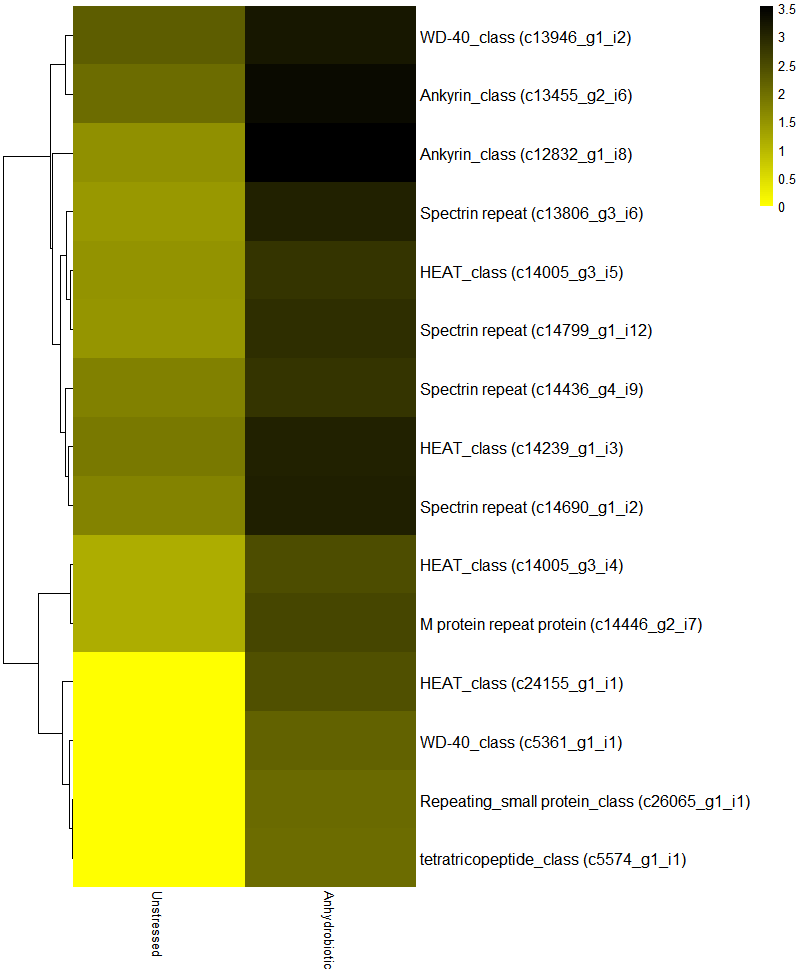

Supplement: S6 Fig — (TIF) [file pone.0275342.s012.tif]

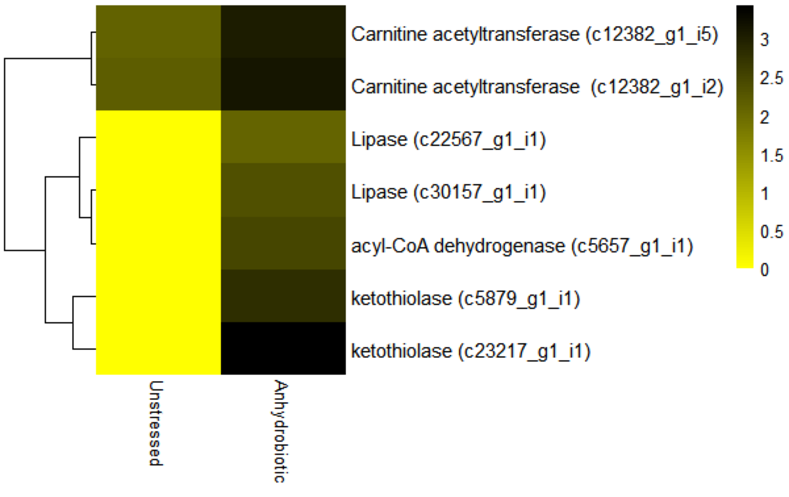

Supplement: S7 Fig — (TIF) [file pone.0275342.s013.tif]

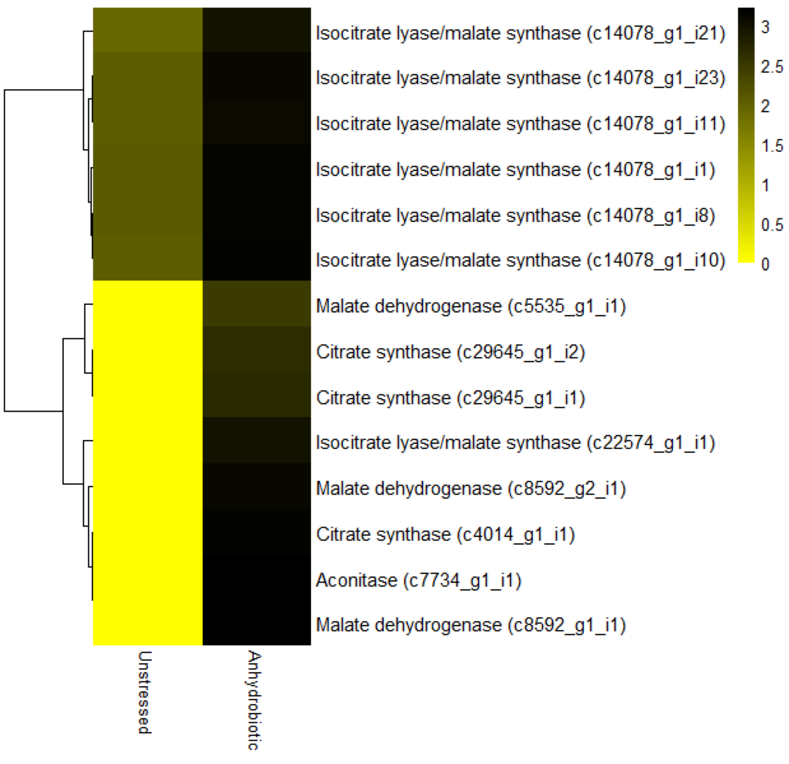

Supplement: S8 Fig — (TIF) [file pone.0275342.s014.tif]

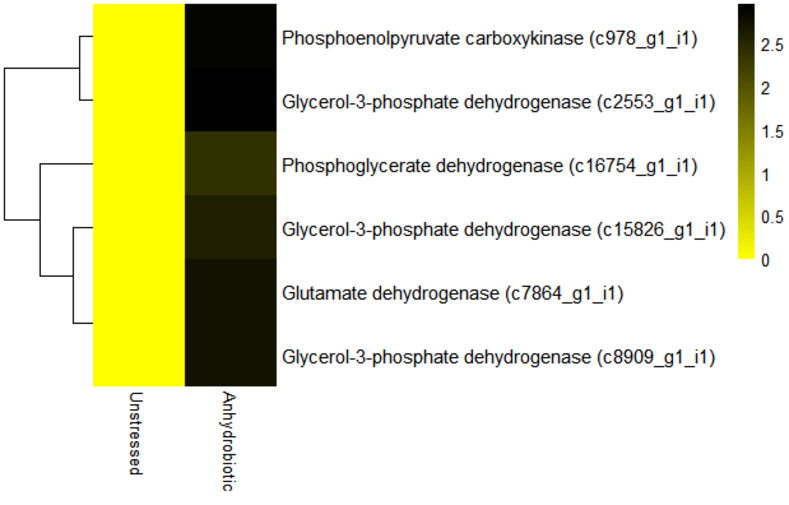

Supplement: S9 Fig — (TIF) [file pone.0275342.s015.tif]

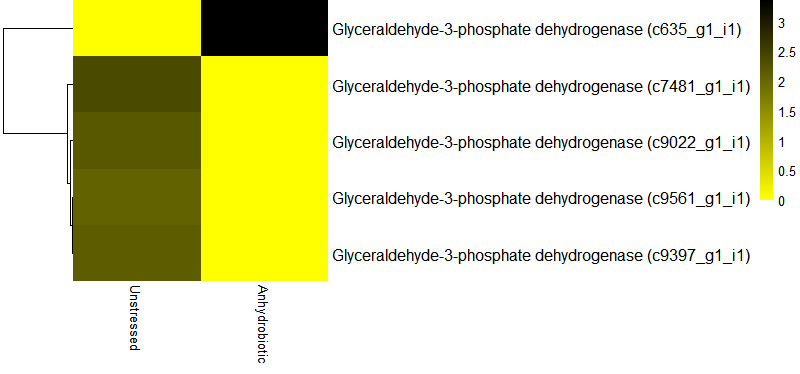

Supplement: S10 Fig — (TIF) [file pone.0275342.s016.tif]

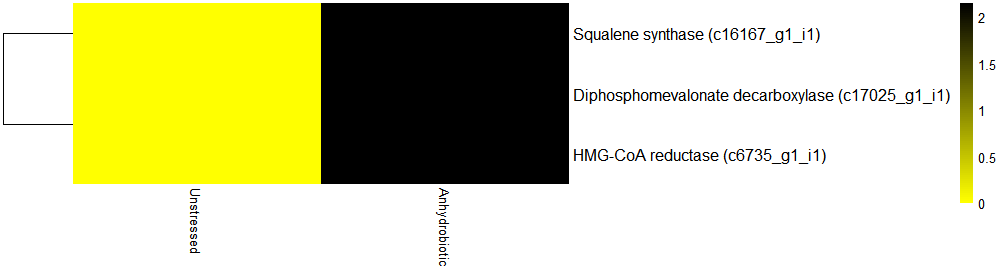

Supplement: S11 Fig — (TIF) [file pone.0275342.s017.tif]

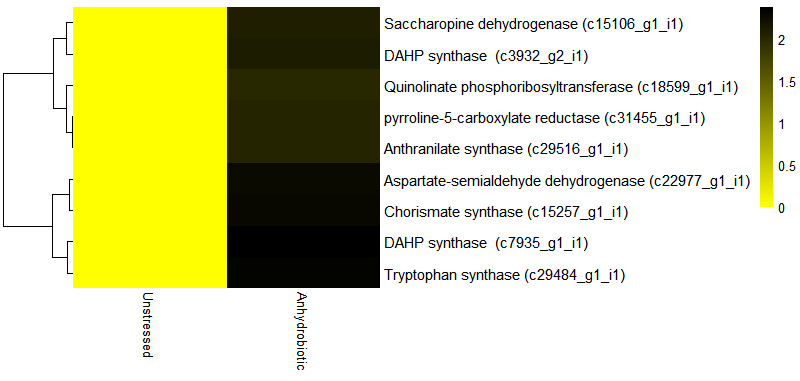

Supplement: S12 Fig — (TIF) [file pone.0275342.s018.tif]

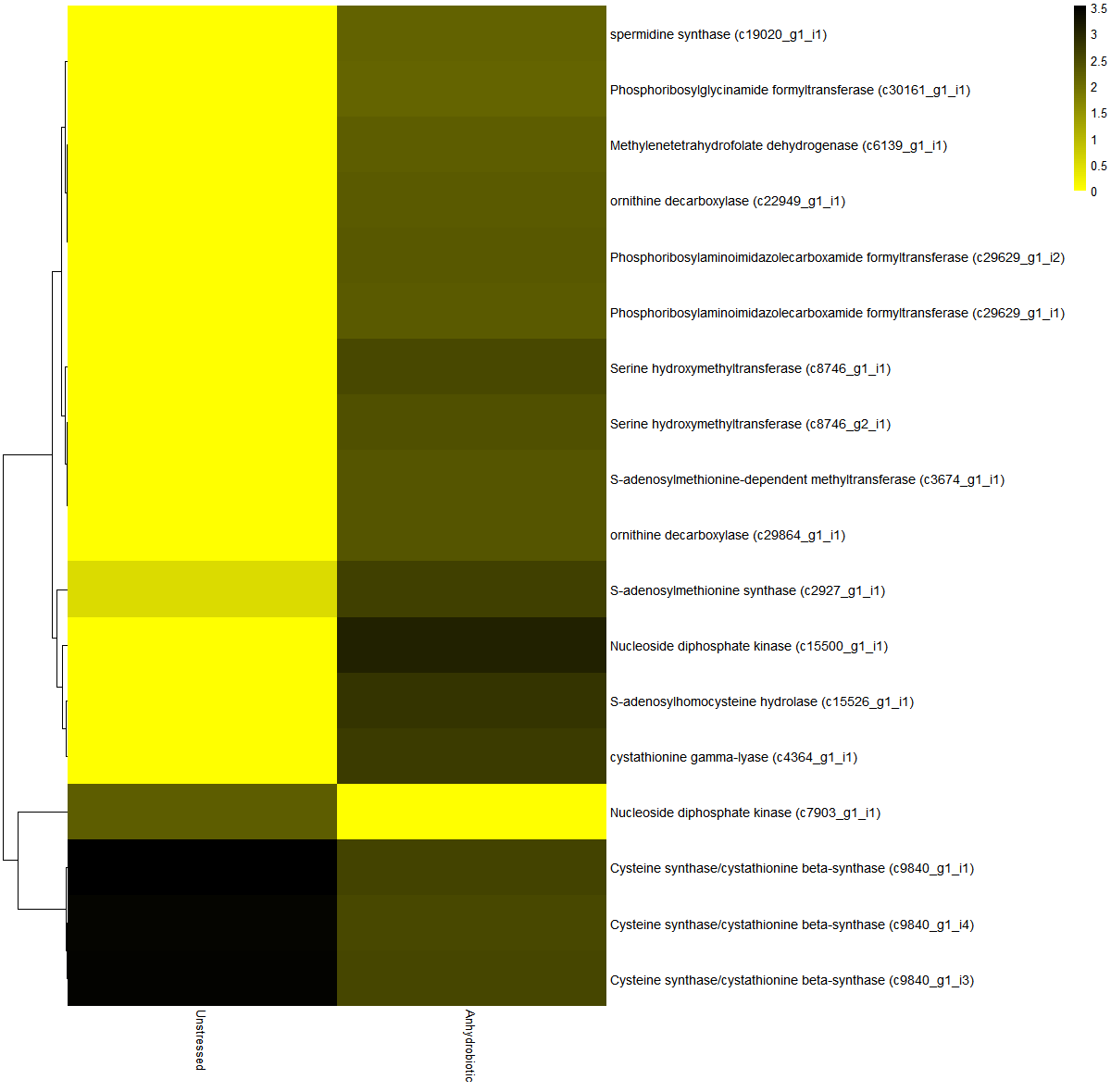

Supplement: S13 Fig — (TIF) [file pone.0275342.s019.tif]
