# Supplementary material for: Deciphering the mechanism of anhydrobiosis in the entomopathogenic nematode Heterorhabditis indica through comparative transcriptomics
Source: PLoS One. 2022 Oct 27;17(10):e0275342. doi: 10.1371/journal.pone.0275342 (PMC9612587; doi:10.1371/journal.pone.0275342)
Supplement: S1 Table — A. Raw read summary. B Trimmed read summary. (DOCX) [file pone.0275342.s020.docx]

**S1A Table. Raw read summary**

| **Sample** | **Read orientation** | **Mean read quality (Phred score)** | **Number of reads** | **% GC** | **% Q > 30** | **Number of bases (MB)** | **Mean read length (bp)** |
| --- | --- | --- | --- | --- | --- | --- | --- |
| Unstressed | R1 | 34.37 | 60589834 | 41.86 | 85.86 | 4887.92 | 80.67 |
|  | R2 | 32.55 | 60589834 | 41.68 | 80.82 | 4891.48 | 80.73 |
| Anhydrobiotic | R1 | 35.57 | 51151316 | 40.18 | 89.77 | 4134.23 | 80.82 |
|  | R2 | 34.47 | 51151316 | 40.00 | 86.94 | 4184.39 | 81.8 |

**S1B Table. Trimmed read summary**

|  | **Unstressed** | **Anhydrobiotic** |
| --- | --- | --- |
| Number of paired-end reads (trimmed) | 60589834 | 51151316 |
| Number of bases (Gb) | 12.12 | 10.23 |
| GC % | 41.97 | 40.29 |
