# Supplementary material for: Deciphering the mechanism of anhydrobiosis in the entomopathogenic nematode Heterorhabditis indica through comparative transcriptomics
Source: PLoS One. 2022 Oct 27;17(10):e0275342. doi: 10.1371/journal.pone.0275342 (PMC9612587; doi:10.1371/journal.pone.0275342)
Supplement: S2 Table — (DOCX) [file pone.0275342.s021.docx]

**S2 Table. Assembled transcript summary of *H. indica* transcriptome**

|  | **All assembled transcripts** | **Transcripts of length**  **≥ 200 bp** |
| --- | --- | --- |
| **Number of assembled transcripts** | 93,932 | 93,932 |
| **Longest transcript length (bp)** | 22,658 | 22,658 |
