# Supplementary material for: Deciphering the mechanism of anhydrobiosis in the entomopathogenic nematode Heterorhabditis indica through comparative transcriptomics
Source: PLoS One. 2022 Oct 27;17(10):e0275342. doi: 10.1371/journal.pone.0275342 (PMC9612587; doi:10.1371/journal.pone.0275342)
Supplement: S3 Table — (DOCX) [file pone.0275342.s022.docx]

**S3 Table. *TransRate* results of *H. indica* transcriptome assembly**

| **Parameters** | **Metrics** |
| --- | --- |
| Assembly | Trinity.fasta |
| n_seqs | 93932 |
| Smallest | 201 |
| Largest | 22658 |
| n_bases | 153050846 |
| mean_len | 1629.37919 |
| n_under_200 | 0 |
| n_over_1k | 47446 |
| n_over_10k | 410 |
| n_with_orf | 40176 |
| mean_orf_percent | 36.79674 |
| n90 | 764 |
| n70 | 1892 |
| n50 | 2843 |
| n30 | 4158 |
| n10 | 6964 |
| Gc | 0.36995 |
| gc_skew | 0.00161 |
| at_skew | 0.0024 |
| cpg_ratio | 1.85865 |
| bases_n | 0 |
| proportion_n | 0 |
| linguistic_complexity | 0.24859 |
