# Supplementary material for: Deciphering the mechanism of anhydrobiosis in the entomopathogenic nematode Heterorhabditis indica through comparative transcriptomics
Source: PLoS One. 2022 Oct 27;17(10):e0275342. doi: 10.1371/journal.pone.0275342 (PMC9612587; doi:10.1371/journal.pone.0275342)
Supplement: S4 Table — (DOCX) [file pone.0275342.s023.docx]

**S4 Table. CEGMA results of *H. indica* transcriptome assembly**

Details of Complete CEG

|  | **Number of 248 ultra-conserved CEGs present in Transcriptome** | **Completeness (%)** | **Total number of CEGs present including putative orthologs** | **Average number of orthologs per CEG** | **percentage of detected CEGs that have more than 1 ortholog** |
| --- | --- | --- | --- | --- | --- |
| Group 1 | 61 | 92.42 | 219 | 3.59 | 83.61 |
| Group 2 | 48 | 85.71 | 184 | 3.83 | 89.58 |
| Group 3 | 58 | 95.08 | 226 | 3.90 | 94.83 |
| Group 4 | 63 | 96.92 | 221 | 3.51 | 85.71 |
| **Total** | **230** | **92.74** | **850** | **3.70** | **88.26** |

Details of partial CEG

|  | **Number of 248 ultra-conserved CEGs present in Transcriptome** | **Completeness (%)** | **Total number of CEGs present including putative orthologs** | **Average number of orthologs per CEG** | **percentage of detected CEGs that have more than 1 ortholog** |
| --- | --- | --- | --- | --- | --- |
| Group 1 | 65 | 98.48 | 272 | 4.18 | 95.38 |
| Group 2 | 53 | 94.64 | 227 | 4.28 | 92.45 |
| Group 3 | 59 | 96.72 | 250 | 4.24 | 93.22 |
| Group 4 | 64 | 98.46 | 275 | 4.30 | 93.75 |
| **Total** | **241** | **97.18** | **1024** | **4.25** | **93.78** |
