# Supplementary material for: Deciphering the mechanism of anhydrobiosis in the entomopathogenic nematode Heterorhabditis indica through comparative transcriptomics
Source: PLoS One. 2022 Oct 27;17(10):e0275342. doi: 10.1371/journal.pone.0275342 (PMC9612587; doi:10.1371/journal.pone.0275342)
Supplement: S5 Table — A. BUSCO results of H. indica transcriptome against Eukaryota protein sets. B. BUSCO results of H. indica transcriptome against Nematoda protein sets. (DOCX) [file pone.0275342.s024.docx]

**S5A Table. BUSCO results of *H. indica* transcriptome against Eukaryota protein sets**

C: 79.2%, F: 12.2%, M: 8.6%, n: 255

| Complete BUSCOs (C) | 202 |
| --- | --- |
| Fragmented BUSCOs (F) | 31 |
| Missing BUSCOs (M) | 22 |
| Total BUSCO groups searched | 255 |

**S5B Table. BUSCO results of *H. indica* transcriptome against Nematoda protein sets**

C: 72.2%, F: 5.7%, M: 22.1%, n: 3131

| Complete BUSCOs (C) | 2261 |
| --- | --- |
| Fragmented BUSCOs (F) | 177 |
| Missing BUSCOs (M) | 693 |
| Total BUSCO groups searched | 3131 |
