# Supplementary material for: Deciphering the mechanism of anhydrobiosis in the entomopathogenic nematode Heterorhabditis indica through comparative transcriptomics
Source: PLoS One. 2022 Oct 27;17(10):e0275342. doi: 10.1371/journal.pone.0275342 (PMC9612587; doi:10.1371/journal.pone.0275342)
Supplement: S6 Table — (DOCX) [file pone.0275342.s025.docx]

**S6 Table.** **Comparative assemble stats report of fresh + anhydrobiotic *H. indica* and Somvanshi *et. al*., (2016)**

| **Parameter** | **Fresh + anhydrobiotic nematode assembly** | **Somvanshi *et al*., 2016** |
| --- | --- | --- |
| No. of reads | 153050846 | 20749541 |
| No. of transcripts | 93932 | 23827 |
| Avg. Transcript size | 1629.38 | 870.84 |
| Largest transcript size | 22658 | 12876 |
| N50 | 2843 | 1291 |
| N60 | 2355 | 1036 |
| N70 | 1892 | 787 |
| N80 | 1393 | 578 |
| N90 | 764 | 383 |
| N100 | 201 | 100 |
| N_count | 0 | 1670 |
| Gaps | 0 | 66 |
