# Supplementary material for: Deciphering the mechanism of anhydrobiosis in the entomopathogenic nematode Heterorhabditis indica through comparative transcriptomics
Source: PLoS One. 2022 Oct 27;17(10):e0275342. doi: 10.1371/journal.pone.0275342 (PMC9612587; doi:10.1371/journal.pone.0275342)
Supplement: S7 Table — (DOCX) [file pone.0275342.s026.docx]

**S7 Table. Read alignment and expression summary of *H. indica* transcriptome**

|  | **Unstressed IJ** | **Anhydrobiotic IJ** |
| --- | --- | --- |
| **Number of filtered reads (paired-end)** | 60,589,834 | 51,151,316 |
| **Number of reads aligned** | 53,815,890 (88.82%) | 43,084,753 (84.23%) |
| **Number of transcripts with FPKM ≥ 1.0** | 39,996 | 48,007 |
