# Supplementary material for: Deciphering the mechanism of anhydrobiosis in the entomopathogenic nematode Heterorhabditis indica through comparative transcriptomics
Source: PLoS One. 2022 Oct 27;17(10):e0275342. doi: 10.1371/journal.pone.0275342 (PMC9612587; doi:10.1371/journal.pone.0275342)
Supplement: S8 Table — (DOCX) [file pone.0275342.s027.docx]

**S8 Table. Transcript expression and total number of transcripts (length ≥ 200bp) of *H. indica***

| **FPKM** | **Number of transcripts** | |
| --- | --- | --- |
|  | **Unstressed IJ** | **Anhydrobiotic IJ** |
| **1.0 - 2.0** | 14,153 | 14,634 |
| **2.0 - 5.0** | 15,357 | 17,597 |
| **5.0 - 10.0** | 5,773 | 8,341 |
| **10.0 - 20.0** | 2,620 | 4,421 |
| **20.0 - 50.0** | 1,433 | 2,252 |
| **50.0 - 100.0** | 383 | 512 |
| **≥ 100.0** | 277 | 250 |
| **Total** | 39,996 | 48,007 |
