# Supplementary material for: Deciphering the mechanism of anhydrobiosis in the entomopathogenic nematode Heterorhabditis indica through comparative transcriptomics
Source: PLoS One. 2022 Oct 27;17(10):e0275342. doi: 10.1371/journal.pone.0275342 (PMC9612587; doi:10.1371/journal.pone.0275342)
Supplement: S9 Table — (DOCX) [file pone.0275342.s028.docx]

**S9 Table. Gene enrichment analysis of differentially expressed genes in anhydrobiotic *H. indica***

| **GO ID** | **GO Name** | **GO Category** | **FDR** | **P-Value** |
| --- | --- | --- | --- | --- |
| GO:0043604 | amide biosynthetic process | BIOLOGICAL_PROCESS | 0.00 | 0.00 |
| GO:0043043 | peptide biosynthetic process | BIOLOGICAL_PROCESS | 0.00 | 0.00 |
| GO:0006412 | translation | BIOLOGICAL_PROCESS | 0.00 | 0.00 |
| GO:0043603 | cellular amide metabolic process | BIOLOGICAL_PROCESS | 0.00 | 0.00 |
| GO:0006518 | peptide metabolic process | BIOLOGICAL_PROCESS | 0.00 | 0.00 |
| GO:0005840 | ribosome | CELLULAR_COMPONENT | 0.00 | 0.00 |
| GO:0034645 | cellular macromolecule biosynthetic process | BIOLOGICAL_PROCESS | 0.00 | 0.00 |
| GO:1901566 | organonitrogen compound biosynthetic process | BIOLOGICAL_PROCESS | 0.00 | 0.00 |
| GO:0003735 | structural constituent of ribosome | MOLECULAR_FUNCTION | 0.00 | 0.00 |
| GO:1901564 | organonitrogen compound metabolic process | BIOLOGICAL_PROCESS | 0.00 | 0.00 |
| GO:0043228 | non-membrane-bounded organelle | CELLULAR_COMPONENT | 0.00 | 0.00 |
| GO:0043232 | intracellular non-membrane-bounded organelle | CELLULAR_COMPONENT | 0.00 | 0.00 |
| GO:0008152 | metabolic process | BIOLOGICAL_PROCESS | 0.00 | 0.00 |
| GO:0044271 | cellular nitrogen compound biosynthetic process | BIOLOGICAL_PROCESS | 0.00 | 0.00 |
| GO:0005198 | structural molecule activity | MOLECULAR_FUNCTION | 0.00 | 0.00 |
| GO:0005622 | intracellular anatomical structure | CELLULAR_COMPONENT | 0.00 | 0.00 |
| GO:1901576 | organic substance biosynthetic process | BIOLOGICAL_PROCESS | 0.00 | 0.00 |
| GO:0019538 | protein metabolic process | BIOLOGICAL_PROCESS | 0.00 | 0.00 |
| GO:0044237 | cellular metabolic process | BIOLOGICAL_PROCESS | 0.00 | 0.00 |
| GO:0009058 | biosynthetic process | BIOLOGICAL_PROCESS | 0.00 | 0.00 |
| GO:0044260 | cellular macromolecule metabolic process | BIOLOGICAL_PROCESS | 0.00 | 0.00 |
| GO:0044249 | cellular biosynthetic process | BIOLOGICAL_PROCESS | 0.00 | 0.00 |
| GO:0022626 | cytosolic ribosome | CELLULAR_COMPONENT | 0.00 | 0.00 |
| GO:0009059 | macromolecule biosynthetic process | BIOLOGICAL_PROCESS | 0.00 | 0.00 |
| GO:0005737 | cytoplasm | CELLULAR_COMPONENT | 0.00 | 0.00 |
| GO:0071704 | organic substance metabolic process | BIOLOGICAL_PROCESS | 0.00 | 0.00 |
| GO:0044238 | primary metabolic process | BIOLOGICAL_PROCESS | 0.00 | 0.00 |
| GO:0044391 | ribosomal subunit | CELLULAR_COMPONENT | 0.00 | 0.00 |
| GO:0010467 | gene expression | BIOLOGICAL_PROCESS | 0.00 | 0.00 |
| GO:0009987 | cellular process | BIOLOGICAL_PROCESS | 0.00 | 0.00 |
| GO:0006807 | nitrogen compound metabolic process | BIOLOGICAL_PROCESS | 0.00 | 0.00 |
| GO:0032991 | protein-containing complex | CELLULAR_COMPONENT | 0.00 | 0.00 |
| GO:0005829 | cytosol | CELLULAR_COMPONENT | 0.00 | 0.00 |
| GO:0034641 | cellular nitrogen compound metabolic process | BIOLOGICAL_PROCESS | 0.00 | 0.00 |
| GO:0043229 | intracellular organelle | CELLULAR_COMPONENT | 0.00 | 0.00 |
| GO:0009277 | fungal-type cell wall | CELLULAR_COMPONENT | 0.00 | 0.00 |
| GO:0043170 | macromolecule metabolic process | BIOLOGICAL_PROCESS | 0.00 | 0.00 |
| GO:0005618 | cell wall | CELLULAR_COMPONENT | 0.00 | 0.00 |
| GO:0043226 | organelle | CELLULAR_COMPONENT | 0.00 | 0.00 |
| GO:0005488 | binding | MOLECULAR_FUNCTION | 0.00 | 0.00 |
| GO:1990904 | ribonucleoprotein complex | CELLULAR_COMPONENT | 0.00 | 0.00 |
| GO:0071840 | cellular component organization or biogenesis | BIOLOGICAL_PROCESS | 0.00 | 0.00 |
| GO:0030446 | hyphal cell wall | CELLULAR_COMPONENT | 0.00 | 0.00 |
| GO:1901363 | heterocyclic compound binding | MOLECULAR_FUNCTION | 0.00 | 0.00 |
| GO:0097159 | organic cyclic compound binding | MOLECULAR_FUNCTION | 0.00 | 0.00 |
| GO:0022625 | cytosolic large ribosomal subunit | CELLULAR_COMPONENT | 0.00 | 0.00 |
| GO:0022627 | cytosolic small ribosomal subunit | CELLULAR_COMPONENT | 0.00 | 0.00 |
| GO:0044085 | cellular component biogenesis | BIOLOGICAL_PROCESS | 0.00 | 0.00 |
| GO:0015935 | small ribosomal subunit | CELLULAR_COMPONENT | 0.00 | 0.00 |
| GO:0015934 | large ribosomal subunit | CELLULAR_COMPONENT | 0.00 | 0.00 |
| GO:0002181 | cytoplasmic translation | BIOLOGICAL_PROCESS | 0.00 | 0.00 |
| GO:0030312 | external encapsulating structure | CELLULAR_COMPONENT | 0.00 | 0.00 |
| GO:0110165 | cellular anatomical entity | CELLULAR_COMPONENT | 0.00 | 0.00 |
| GO:0022613 | ribonucleoprotein complex biogenesis | BIOLOGICAL_PROCESS | 0.00 | 0.00 |
| GO:0030445 | yeast-form cell wall | CELLULAR_COMPONENT | 0.00 | 0.00 |
| GO:0042254 | ribosome biogenesis | BIOLOGICAL_PROCESS | 0.00 | 0.00 |
| GO:0062039 | biofilm matrix | CELLULAR_COMPONENT | 0.00 | 0.00 |
| GO:0062040 | fungal biofilm matrix | CELLULAR_COMPONENT | 0.00 | 0.00 |
| GO:0003723 | RNA binding | MOLECULAR_FUNCTION | 0.00 | 0.00 |
| GO:0071554 | cell wall organization or biogenesis | BIOLOGICAL_PROCESS | 0.00 | 0.00 |
| GO:0044281 | small molecule metabolic process | BIOLOGICAL_PROCESS | 0.00 | 0.00 |
| GO:0006996 | organelle organization | BIOLOGICAL_PROCESS | 0.00 | 0.00 |
| GO:0016043 | cellular component organization | BIOLOGICAL_PROCESS | 0.00 | 0.00 |
| GO:0044283 | small molecule biosynthetic process | BIOLOGICAL_PROCESS | 0.00 | 0.00 |
| GO:0009986 | cell surface | CELLULAR_COMPONENT | 0.00 | 0.00 |
| GO:0097367 | carbohydrate derivative binding | MOLECULAR_FUNCTION | 0.00 | 0.00 |
| GO:0006091 | generation of precursor metabolites and energy | BIOLOGICAL_PROCESS | 0.00 | 0.00 |
| GO:0003824 | catalytic activity | MOLECULAR_FUNCTION | 0.00 | 0.00 |
| GO:0036094 | small molecule binding | MOLECULAR_FUNCTION | 0.00 | 0.00 |
| GO:1901265 | nucleoside phosphate binding | MOLECULAR_FUNCTION | 0.00 | 0.00 |
| GO:0000166 | nucleotide binding | MOLECULAR_FUNCTION | 0.00 | 0.00 |
| GO:0071852 | fungal-type cell wall organization or biogenesis | BIOLOGICAL_PROCESS | 0.00 | 0.00 |
| GO:0035639 | purine ribonucleoside triphosphate binding | MOLECULAR_FUNCTION | 0.00 | 0.00 |
| GO:0030554 | adenyl nucleotide binding | MOLECULAR_FUNCTION | 0.00 | 0.00 |
| GO:0005524 | ATP binding | MOLECULAR_FUNCTION | 0.00 | 0.00 |
| GO:0032559 | adenyl ribonucleotide binding | MOLECULAR_FUNCTION | 0.00 | 0.00 |
| GO:0065010 | extracellular membrane-bounded organelle | CELLULAR_COMPONENT | 0.00 | 0.00 |
| GO:1903561 | extracellular vesicle | CELLULAR_COMPONENT | 0.00 | 0.00 |
| GO:0043230 | extracellular organelle | CELLULAR_COMPONENT | 0.00 | 0.00 |
| GO:0017076 | purine nucleotide binding | MOLECULAR_FUNCTION | 0.00 | 0.00 |
| GO:0032553 | ribonucleotide binding | MOLECULAR_FUNCTION | 0.00 | 0.00 |
| GO:0032555 | purine ribonucleotide binding | MOLECULAR_FUNCTION | 0.00 | 0.00 |
| GO:0016072 | rRNA metabolic process | BIOLOGICAL_PROCESS | 0.00 | 0.00 |
| GO:0006696 | ergosterol biosynthetic process | BIOLOGICAL_PROCESS | 0.00 | 0.00 |
| GO:0044108 | cellular alcohol biosynthetic process | BIOLOGICAL_PROCESS | 0.00 | 0.00 |
| GO:0043168 | anion binding | MOLECULAR_FUNCTION | 0.00 | 0.00 |
| GO:0044107 | cellular alcohol metabolic process | BIOLOGICAL_PROCESS | 0.00 | 0.00 |
| GO:0008204 | ergosterol metabolic process | BIOLOGICAL_PROCESS | 0.00 | 0.00 |
| GO:0043436 | oxoacid metabolic process | BIOLOGICAL_PROCESS | 0.00 | 0.00 |
| GO:0006082 | organic acid metabolic process | BIOLOGICAL_PROCESS | 0.00 | 0.00 |
| GO:0043167 | ion binding | MOLECULAR_FUNCTION | 0.00 | 0.00 |
| GO:0016129 | phytosteroid biosynthetic process | BIOLOGICAL_PROCESS | 0.00 | 0.00 |
| GO:0019752 | carboxylic acid metabolic process | BIOLOGICAL_PROCESS | 0.00 | 0.00 |
| GO:0097384 | cellular lipid biosynthetic process | BIOLOGICAL_PROCESS | 0.00 | 0.00 |
| GO:0009056 | catabolic process | BIOLOGICAL_PROCESS | 0.00 | 0.00 |
| GO:0016128 | phytosteroid metabolic process | BIOLOGICAL_PROCESS | 0.00 | 0.00 |
| GO:0000785 | chromatin | CELLULAR_COMPONENT | 0.00 | 0.00 |
| GO:0016126 | sterol biosynthetic process | BIOLOGICAL_PROCESS | 0.00 | 0.00 |
| GO:0045333 | cellular respiration | BIOLOGICAL_PROCESS | 0.00 | 0.00 |
| GO:0042546 | cell wall biogenesis | BIOLOGICAL_PROCESS | 0.00 | 0.00 |
| GO:1902653 | secondary alcohol biosynthetic process | BIOLOGICAL_PROCESS | 0.00 | 0.00 |
| GO:0019843 | rRNA binding | MOLECULAR_FUNCTION | 0.00 | 0.00 |
| GO:0005887 | integral component of plasma membrane | CELLULAR_COMPONENT | 0.00 | 0.00 |
| GO:0044248 | cellular catabolic process | BIOLOGICAL_PROCESS | 0.00 | 0.00 |
| GO:0072347 | response to anesthetic | BIOLOGICAL_PROCESS | 0.00 | 0.00 |
| GO:0022607 | cellular component assembly | BIOLOGICAL_PROCESS | 0.00 | 0.00 |
| GO:0006364 | rRNA processing | BIOLOGICAL_PROCESS | 0.00 | 0.00 |
| GO:0015980 | energy derivation by oxidation of organic compounds | BIOLOGICAL_PROCESS | 0.00 | 0.00 |
| GO:0000462 | maturation of SSU-rRNA from tricistronic rRNA transcript (SSU-rRNA, 5.8S rRNA, LSU-rRNA) | BIOLOGICAL_PROCESS | 0.00 | 0.00 |
| GO:0008652 | cellular amino acid biosynthetic process | BIOLOGICAL_PROCESS | 0.00 | 0.00 |
| GO:0006694 | steroid biosynthetic process | BIOLOGICAL_PROCESS | 0.00 | 0.00 |
| GO:0030490 | maturation of SSU-rRNA | BIOLOGICAL_PROCESS | 0.00 | 0.00 |
| GO:0031505 | fungal-type cell wall organization | BIOLOGICAL_PROCESS | 0.00 | 0.00 |
| GO:0042273 | ribosomal large subunit biogenesis | BIOLOGICAL_PROCESS | 0.00 | 0.00 |
| GO:0030447 | filamentous growth | BIOLOGICAL_PROCESS | 0.00 | 0.00 |
| GO:0042274 | ribosomal small subunit biogenesis | BIOLOGICAL_PROCESS | 0.00 | 0.00 |
| GO:0009272 | fungal-type cell wall biogenesis | BIOLOGICAL_PROCESS | 0.00 | 0.00 |
| GO:0030234 | enzyme regulator activity | MOLECULAR_FUNCTION | 0.00 | 0.00 |
| GO:0071555 | cell wall organization | BIOLOGICAL_PROCESS | 0.00 | 0.00 |
| GO:0003676 | nucleic acid binding | MOLECULAR_FUNCTION | 0.00 | 0.00 |
| GO:0043933 | protein-containing complex organization | BIOLOGICAL_PROCESS | 0.00 | 0.00 |
| GO:0060089 | molecular transducer activity | MOLECULAR_FUNCTION | 0.00 | 0.00 |
| GO:0046165 | alcohol biosynthetic process | BIOLOGICAL_PROCESS | 0.00 | 0.00 |
| GO:0004857 | enzyme inhibitor activity | MOLECULAR_FUNCTION | 0.00 | 0.00 |
| GO:1901617 | organic hydroxy compound biosynthetic process | BIOLOGICAL_PROCESS | 0.00 | 0.00 |
| GO:0004066 | asparagine synthase (glutamine-hydrolyzing) activity | MOLECULAR_FUNCTION | 0.00 | 0.00 |
| GO:0006529 | asparagine biosynthetic process | BIOLOGICAL_PROCESS | 0.00 | 0.00 |
| GO:0038023 | signaling receptor activity | MOLECULAR_FUNCTION | 0.00 | 0.00 |
| GO:0098772 | molecular function regulator activity | MOLECULAR_FUNCTION | 0.00 | 0.00 |
| GO:0022618 | ribonucleoprotein complex assembly | BIOLOGICAL_PROCESS | 0.00 | 0.00 |
| GO:1902652 | secondary alcohol metabolic process | BIOLOGICAL_PROCESS | 0.00 | 0.00 |
| GO:0016125 | sterol metabolic process | BIOLOGICAL_PROCESS | 0.00 | 0.00 |
| GO:0051246 | regulation of protein metabolic process | BIOLOGICAL_PROCESS | 0.00 | 0.00 |
| GO:0070981 | L-asparagine biosynthetic process | BIOLOGICAL_PROCESS | 0.00 | 0.00 |
| GO:0070982 | L-asparagine metabolic process | BIOLOGICAL_PROCESS | 0.00 | 0.00 |
| GO:0051248 | negative regulation of protein metabolic process | BIOLOGICAL_PROCESS | 0.00 | 0.00 |
| GO:0006508 | proteolysis | BIOLOGICAL_PROCESS | 0.00 | 0.00 |
| GO:0046394 | carboxylic acid biosynthetic process | BIOLOGICAL_PROCESS | 0.00 | 0.00 |
| GO:0071826 | ribonucleoprotein complex subunit organization | BIOLOGICAL_PROCESS | 0.00 | 0.00 |
| GO:0043231 | intracellular membrane-bounded organelle | CELLULAR_COMPONENT | 0.00 | 0.00 |
| GO:0006528 | asparagine metabolic process | BIOLOGICAL_PROCESS | 0.00 | 0.00 |
| GO:0043291 | RAVE complex | CELLULAR_COMPONENT | 0.00 | 0.00 |
| GO:0006520 | cellular amino acid metabolic process | BIOLOGICAL_PROCESS | 0.00 | 0.00 |
| GO:0004930 | G protein-coupled receptor activity | MOLECULAR_FUNCTION | 0.00 | 0.00 |
| GO:0044092 | negative regulation of molecular function | BIOLOGICAL_PROCESS | 0.00 | 0.00 |
| GO:0008643 | carbohydrate transport | BIOLOGICAL_PROCESS | 0.00 | 0.00 |
| GO:0009060 | aerobic respiration | BIOLOGICAL_PROCESS | 0.00 | 0.00 |
| GO:0043086 | negative regulation of catalytic activity | BIOLOGICAL_PROCESS | 0.00 | 0.00 |
| GO:0004888 | transmembrane signaling receptor activity | MOLECULAR_FUNCTION | 0.00 | 0.00 |
| GO:0016053 | organic acid biosynthetic process | BIOLOGICAL_PROCESS | 0.00 | 0.00 |
| GO:0005975 | carbohydrate metabolic process | BIOLOGICAL_PROCESS | 0.00 | 0.00 |
| GO:0042255 | ribosome assembly | BIOLOGICAL_PROCESS | 0.00 | 0.00 |
| GO:0051346 | negative regulation of hydrolase activity | BIOLOGICAL_PROCESS | 0.00 | 0.00 |
| GO:1902494 | catalytic complex | CELLULAR_COMPONENT | 0.00 | 0.00 |
| GO:0065003 | protein-containing complex assembly | BIOLOGICAL_PROCESS | 0.00 | 0.00 |
| GO:0009536 | plastid | CELLULAR_COMPONENT | 0.00 | 0.00 |
| GO:0005576 | extracellular region | CELLULAR_COMPONENT | 0.00 | 0.00 |
| GO:0043227 | membrane-bounded organelle | CELLULAR_COMPONENT | 0.00 | 0.00 |
| GO:0031226 | intrinsic component of plasma membrane | CELLULAR_COMPONENT | 0.00 | 0.00 |
| GO:0071702 | organic substance transport | BIOLOGICAL_PROCESS | 0.00 | 0.00 |
| GO:0019222 | regulation of metabolic process | BIOLOGICAL_PROCESS | 0.00 | 0.00 |
| GO:1901607 | alpha-amino acid biosynthetic process | BIOLOGICAL_PROCESS | 0.00 | 0.00 |
| GO:1901575 | organic substance catabolic process | BIOLOGICAL_PROCESS | 0.00 | 0.00 |
| GO:0051172 | negative regulation of nitrogen compound metabolic process | BIOLOGICAL_PROCESS | 0.00 | 0.00 |
| GO:0008202 | steroid metabolic process | BIOLOGICAL_PROCESS | 0.00 | 0.00 |
| GO:0032655 | regulation of interleukin-12 production | BIOLOGICAL_PROCESS | 0.00 | 0.00 |
| GO:0032615 | interleukin-12 production | BIOLOGICAL_PROCESS | 0.00 | 0.00 |
| GO:0006796 | phosphate-containing compound metabolic process | BIOLOGICAL_PROCESS | 0.00 | 0.00 |
| GO:0044038 | cell wall macromolecule biosynthetic process | BIOLOGICAL_PROCESS | 0.00 | 0.00 |
| GO:0070589 | cellular component macromolecule biosynthetic process | BIOLOGICAL_PROCESS | 0.00 | 0.00 |
| GO:0015248 | sterol transporter activity | MOLECULAR_FUNCTION | 0.00 | 0.00 |
| GO:0006793 | phosphorus metabolic process | BIOLOGICAL_PROCESS | 0.00 | 0.00 |
| GO:0043161 | proteasome-mediated ubiquitin-dependent protein catabolic process | BIOLOGICAL_PROCESS | 0.00 | 0.00 |
| GO:0009507 | chloroplast | CELLULAR_COMPONENT | 0.00 | 0.00 |
| GO:1901360 | organic cyclic compound metabolic process | BIOLOGICAL_PROCESS | 0.00 | 0.00 |
| GO:0046982 | protein heterodimerization activity | MOLECULAR_FUNCTION | 0.00 | 0.00 |
| GO:0042592 | homeostatic process | BIOLOGICAL_PROCESS | 0.00 | 0.00 |
| GO:0007035 | vacuolar acidification | BIOLOGICAL_PROCESS | 0.00 | 0.00 |
| GO:0009057 | macromolecule catabolic process | BIOLOGICAL_PROCESS | 0.00 | 0.00 |
| GO:0005694 | chromosome | CELLULAR_COMPONENT | 0.00 | 0.00 |
| GO:1990204 | oxidoreductase complex | CELLULAR_COMPONENT | 0.00 | 0.00 |
| GO:0140694 | non-membrane-bounded organelle assembly | BIOLOGICAL_PROCESS | 0.00 | 0.00 |
| GO:1901362 | organic cyclic compound biosynthetic process | BIOLOGICAL_PROCESS | 0.00 | 0.00 |
| GO:0016236 | macroautophagy | BIOLOGICAL_PROCESS | 0.00 | 0.00 |
| GO:0060255 | regulation of macromolecule metabolic process | BIOLOGICAL_PROCESS | 0.00 | 0.00 |
| GO:0006066 | alcohol metabolic process | BIOLOGICAL_PROCESS | 0.00 | 0.00 |
| GO:0030162 | regulation of proteolysis | BIOLOGICAL_PROCESS | 0.00 | 0.00 |
| GO:0010605 | negative regulation of macromolecule metabolic process | BIOLOGICAL_PROCESS | 0.00 | 0.00 |
| GO:0070013 | intracellular organelle lumen | CELLULAR_COMPONENT | 0.00 | 0.00 |
| GO:0043233 | organelle lumen | CELLULAR_COMPONENT | 0.00 | 0.00 |
| GO:0031974 | membrane-enclosed lumen | CELLULAR_COMPONENT | 0.00 | 0.00 |
| GO:0044036 | cell wall macromolecule metabolic process | BIOLOGICAL_PROCESS | 0.00 | 0.00 |
| GO:0061919 | process utilizing autophagic mechanism | BIOLOGICAL_PROCESS | 0.00 | 0.00 |
| GO:0042123 | glucanosyltransferase activity | MOLECULAR_FUNCTION | 0.00 | 0.00 |
| GO:0042124 | 1,3-beta-glucanosyltransferase activity | MOLECULAR_FUNCTION | 0.00 | 0.00 |
| GO:0007186 | G protein-coupled receptor signaling pathway | BIOLOGICAL_PROCESS | 0.00 | 0.00 |
| GO:0009892 | negative regulation of metabolic process | BIOLOGICAL_PROCESS | 0.00 | 0.00 |
| GO:0034660 | ncRNA metabolic process | BIOLOGICAL_PROCESS | 0.00 | 0.00 |
| GO:0051452 | intracellular pH reduction | BIOLOGICAL_PROCESS | 0.00 | 0.00 |
| GO:0040017 | positive regulation of locomotion | BIOLOGICAL_PROCESS | 0.00 | 0.00 |
| GO:0043632 | modification-dependent macromolecule catabolic process | BIOLOGICAL_PROCESS | 0.00 | 0.00 |
| GO:0033365 | protein localization to organelle | BIOLOGICAL_PROCESS | 0.00 | 0.00 |
| GO:0044182 | filamentous growth of a population of unicellular organisms | BIOLOGICAL_PROCESS | 0.00 | 0.00 |
| GO:0005739 | mitochondrion | CELLULAR_COMPONENT | 0.00 | 0.00 |
| GO:0009896 | positive regulation of catabolic process | BIOLOGICAL_PROCESS | 0.00 | 0.00 |
| GO:0006914 | autophagy | BIOLOGICAL_PROCESS | 0.00 | 0.00 |
| GO:0050790 | regulation of catalytic activity | BIOLOGICAL_PROCESS | 0.00 | 0.00 |
| GO:0019941 | modification-dependent protein catabolic process | BIOLOGICAL_PROCESS | 0.00 | 0.00 |
| GO:0006325 | chromatin organization | BIOLOGICAL_PROCESS | 0.00 | 0.00 |
| GO:0000324 | fungal-type vacuole | CELLULAR_COMPONENT | 0.00 | 0.00 |
| GO:0000322 | storage vacuole | CELLULAR_COMPONENT | 0.00 | 0.00 |
| GO:0042645 | mitochondrial nucleoid | CELLULAR_COMPONENT | 0.00 | 0.00 |
| GO:0065009 | regulation of molecular function | BIOLOGICAL_PROCESS | 0.00 | 0.00 |
| GO:0044265 | cellular macromolecule catabolic process | BIOLOGICAL_PROCESS | 0.00 | 0.00 |
| GO:0031331 | positive regulation of cellular catabolic process | BIOLOGICAL_PROCESS | 0.00 | 0.00 |
| GO:0072594 | establishment of protein localization to organelle | BIOLOGICAL_PROCESS | 0.00 | 0.00 |
| GO:0010498 | proteasomal protein catabolic process | BIOLOGICAL_PROCESS | 0.00 | 0.00 |
| GO:0009295 | nucleoid | CELLULAR_COMPONENT | 0.00 | 0.00 |
| GO:0000786 | nucleosome | CELLULAR_COMPONENT | 0.00 | 0.00 |
| GO:0009303 | rRNA transcription | BIOLOGICAL_PROCESS | 0.00 | 0.00 |
| GO:0044815 | DNA packaging complex | CELLULAR_COMPONENT | 0.00 | 0.00 |
| GO:1901615 | organic hydroxy compound metabolic process | BIOLOGICAL_PROCESS | 0.00 | 0.00 |
| GO:0006099 | tricarboxylic acid cycle | BIOLOGICAL_PROCESS | 0.00 | 0.00 |
| GO:0015918 | sterol transport | BIOLOGICAL_PROCESS | 0.00 | 0.00 |
| GO:0010951 | negative regulation of endopeptidase activity | BIOLOGICAL_PROCESS | 0.00 | 0.00 |
| GO:0030163 | protein catabolic process | BIOLOGICAL_PROCESS | 0.00 | 0.00 |
| GO:0004866 | endopeptidase inhibitor activity | MOLECULAR_FUNCTION | 0.00 | 0.00 |
| GO:0030435 | sporulation resulting in formation of a cellular spore | BIOLOGICAL_PROCESS | 0.00 | 0.00 |
| GO:0043934 | sporulation | BIOLOGICAL_PROCESS | 0.00 | 0.00 |
| GO:1901605 | alpha-amino acid metabolic process | BIOLOGICAL_PROCESS | 0.00 | 0.00 |
| GO:0051336 | regulation of hydrolase activity | BIOLOGICAL_PROCESS | 0.00 | 0.00 |
| GO:0051701 | biological process involved in interaction with host | BIOLOGICAL_PROCESS | 0.00 | 0.00 |
| GO:0070592 | cell wall polysaccharide biosynthetic process | BIOLOGICAL_PROCESS | 0.00 | 0.00 |
| GO:0006511 | ubiquitin-dependent protein catabolic process | BIOLOGICAL_PROCESS | 0.00 | 0.00 |
| GO:0017111 | nucleoside-triphosphatase activity | MOLECULAR_FUNCTION | 0.00 | 0.00 |
| GO:0051603 | proteolysis involved in protein catabolic process | BIOLOGICAL_PROCESS | 0.00 | 0.00 |
| GO:0051276 | chromosome organization | BIOLOGICAL_PROCESS | 0.00 | 0.00 |
| GO:0070603 | SWI/SNF superfamily-type complex | CELLULAR_COMPONENT | 0.00 | 0.00 |
| GO:0016740 | transferase activity | MOLECULAR_FUNCTION | 0.00 | 0.00 |
| GO:0000027 | ribosomal large subunit assembly | BIOLOGICAL_PROCESS | 0.00 | 0.00 |
| GO:0016887 | ATP hydrolysis activity | MOLECULAR_FUNCTION | 0.00 | 0.00 |
| GO:0061135 | endopeptidase regulator activity | MOLECULAR_FUNCTION | 0.00 | 0.00 |
| GO:0044403 | biological process involved in symbiotic interaction | BIOLOGICAL_PROCESS | 0.00 | 0.00 |
| GO:0052548 | regulation of endopeptidase activity | BIOLOGICAL_PROCESS | 0.00 | 0.00 |
| GO:0006897 | endocytosis | BIOLOGICAL_PROCESS | 0.00 | 0.00 |
| GO:0061912 | selective autophagy | BIOLOGICAL_PROCESS | 0.00 | 0.00 |
| GO:0016462 | pyrophosphatase activity | MOLECULAR_FUNCTION | 0.00 | 0.00 |
| GO:0006725 | cellular aromatic compound metabolic process | BIOLOGICAL_PROCESS | 0.00 | 0.00 |
| GO:0048518 | positive regulation of biological process | BIOLOGICAL_PROCESS | 0.00 | 0.00 |
| GO:0010383 | cell wall polysaccharide metabolic process | BIOLOGICAL_PROCESS | 0.00 | 0.00 |
| GO:0042395 | ecdysis, collagen and cuticulin-based cuticle | BIOLOGICAL_PROCESS | 0.00 | 0.00 |
| GO:0070925 | organelle assembly | BIOLOGICAL_PROCESS | 0.00 | 0.00 |
| GO:0030641 | regulation of cellular pH | BIOLOGICAL_PROCESS | 0.00 | 0.00 |
| GO:0005905 | clathrin-coated pit | CELLULAR_COMPONENT | 0.00 | 0.00 |
| GO:0051453 | regulation of intracellular pH | BIOLOGICAL_PROCESS | 0.00 | 0.00 |
| GO:0008135 | translation factor activity, RNA binding | MOLECULAR_FUNCTION | 0.00 | 0.00 |
| GO:0140657 | ATP-dependent activity | MOLECULAR_FUNCTION | 0.00 | 0.00 |
| GO:0046483 | heterocycle metabolic process | BIOLOGICAL_PROCESS | 0.00 | 0.00 |
| GO:1904949 | ATPase complex | CELLULAR_COMPONENT | 0.00 | 0.00 |
| GO:0098798 | mitochondrial protein-containing complex | CELLULAR_COMPONENT | 0.00 | 0.00 |
| GO:0043457 | regulation of cellular respiration | BIOLOGICAL_PROCESS | 0.00 | 0.00 |
| GO:0016818 | hydrolase activity, acting on acid anhydrides, in phosphorus-containing anhydrides | MOLECULAR_FUNCTION | 0.00 | 0.00 |
| GO:0009894 | regulation of catabolic process | BIOLOGICAL_PROCESS | 0.00 | 0.00 |
| GO:0016817 | hydrolase activity, acting on acid anhydrides | MOLECULAR_FUNCTION | 0.00 | 0.00 |
| GO:1901565 | organonitrogen compound catabolic process | BIOLOGICAL_PROCESS | 0.00 | 0.00 |
| GO:0031225 | anchored component of membrane | CELLULAR_COMPONENT | 0.00 | 0.00 |
| GO:0004713 | protein tyrosine kinase activity | MOLECULAR_FUNCTION | 0.00 | 0.00 |
| GO:0004867 | serine-type endopeptidase inhibitor activity | MOLECULAR_FUNCTION | 0.00 | 0.00 |
| GO:0046912 | acyltransferase activity, acyl groups converted into alkyl on transfer | MOLECULAR_FUNCTION | 0.00 | 0.00 |
| GO:0005095 | GTPase inhibitor activity | MOLECULAR_FUNCTION | 0.00 | 0.00 |
| GO:1902634 | 1-phosphatidyl-1D-myo-inositol 4,5-bisphosphate catabolic process | BIOLOGICAL_PROCESS | 0.00 | 0.00 |
| GO:0032126 | eisosome | CELLULAR_COMPONENT | 0.00 | 0.00 |
| GO:0016310 | phosphorylation | BIOLOGICAL_PROCESS | 0.00 | 0.00 |
| GO:0006363 | termination of RNA polymerase I transcription | BIOLOGICAL_PROCESS | 0.00 | 0.00 |
| GO:0030863 | cortical cytoskeleton | CELLULAR_COMPONENT | 0.00 | 0.00 |
| GO:0022404 | molting cycle process | BIOLOGICAL_PROCESS | 0.00 | 0.00 |
| GO:0030427 | site of polarized growth | CELLULAR_COMPONENT | 0.00 | 0.00 |
| GO:0090079 | translation regulator activity, nucleic acid binding | MOLECULAR_FUNCTION | 0.00 | 0.00 |
| GO:0010466 | negative regulation of peptidase activity | BIOLOGICAL_PROCESS | 0.00 | 0.00 |
| GO:0009410 | response to xenobiotic stimulus | BIOLOGICAL_PROCESS | 0.00 | 0.00 |
| GO:0045182 | translation regulator activity | MOLECULAR_FUNCTION | 0.00 | 0.00 |
| GO:0006139 | nucleobase-containing compound metabolic process | BIOLOGICAL_PROCESS | 0.00 | 0.00 |
| GO:0016884 | carbon-nitrogen ligase activity, with glutamine as amido-N-donor | MOLECULAR_FUNCTION | 0.00 | 0.00 |
| GO:0051274 | beta-glucan biosynthetic process | BIOLOGICAL_PROCESS | 0.00 | 0.00 |
| GO:0033554 | cellular response to stress | BIOLOGICAL_PROCESS | 0.00 | 0.00 |
| GO:0005933 | cellular bud | CELLULAR_COMPONENT | 0.00 | 0.00 |
| GO:0034599 | cellular response to oxidative stress | BIOLOGICAL_PROCESS | 0.00 | 0.00 |
| GO:0015399 | primary active transmembrane transporter activity | MOLECULAR_FUNCTION | 0.00 | 0.00 |
| GO:0016491 | oxidoreductase activity | MOLECULAR_FUNCTION | 0.00 | 0.00 |
| GO:0010499 | proteasomal ubiquitin-independent protein catabolic process | BIOLOGICAL_PROCESS | 0.00 | 0.00 |
| GO:0006362 | transcription elongation from RNA polymerase I promoter | BIOLOGICAL_PROCESS | 0.00 | 0.00 |
| GO:0062197 | cellular response to chemical stress | BIOLOGICAL_PROCESS | 0.00 | 0.00 |
| GO:0034293 | sexual sporulation | BIOLOGICAL_PROCESS | 0.00 | 0.00 |
| GO:0043935 | sexual sporulation resulting in formation of a cellular spore | BIOLOGICAL_PROCESS | 0.00 | 0.00 |
| GO:0015977 | carbon fixation | BIOLOGICAL_PROCESS | 0.00 | 0.00 |
| GO:0019685 | photosynthesis, dark reaction | BIOLOGICAL_PROCESS | 0.00 | 0.00 |
| GO:0019253 | reductive pentose-phosphate cycle | BIOLOGICAL_PROCESS | 0.00 | 0.00 |
| GO:0018108 | peptidyl-tyrosine phosphorylation | BIOLOGICAL_PROCESS | 0.00 | 0.00 |
| GO:0006885 | regulation of pH | BIOLOGICAL_PROCESS | 0.00 | 0.00 |
| GO:0005515 | protein binding | MOLECULAR_FUNCTION | 0.00 | 0.00 |
| GO:0006950 | response to stress | BIOLOGICAL_PROCESS | 0.00 | 0.00 |
| GO:0045989 | positive regulation of striated muscle contraction | BIOLOGICAL_PROCESS | 0.00 | 0.00 |
| GO:0045861 | negative regulation of proteolysis | BIOLOGICAL_PROCESS | 0.00 | 0.00 |
| GO:0018212 | peptidyl-tyrosine modification | BIOLOGICAL_PROCESS | 0.00 | 0.00 |
| GO:0051273 | beta-glucan metabolic process | BIOLOGICAL_PROCESS | 0.00 | 0.00 |
| GO:0031329 | regulation of cellular catabolic process | BIOLOGICAL_PROCESS | 0.00 | 0.00 |
| GO:0061134 | peptidase regulator activity | MOLECULAR_FUNCTION | 0.00 | 0.00 |
| GO:0016237 | lysosomal microautophagy | BIOLOGICAL_PROCESS | 0.00 | 0.00 |
| GO:0030414 | peptidase inhibitor activity | MOLECULAR_FUNCTION | 0.00 | 0.00 |
| GO:0009067 | aspartate family amino acid biosynthetic process | BIOLOGICAL_PROCESS | 0.00 | 0.00 |
| GO:0002183 | cytoplasmic translational initiation | BIOLOGICAL_PROCESS | 0.00 | 0.00 |
| GO:0000470 | maturation of LSU-rRNA | BIOLOGICAL_PROCESS | 0.00 | 0.00 |
| GO:0022411 | cellular component disassembly | BIOLOGICAL_PROCESS | 0.00 | 0.00 |
| GO:0006813 | potassium ion transport | BIOLOGICAL_PROCESS | 0.00 | 0.00 |
| GO:0015979 | photosynthesis | BIOLOGICAL_PROCESS | 0.00 | 0.00 |
| GO:0061638 | CENP-A containing chromatin | CELLULAR_COMPONENT | 0.00 | 0.00 |
| GO:0006075 | (1->3)-beta-D-glucan biosynthetic process | BIOLOGICAL_PROCESS | 0.00 | 0.00 |
| GO:0006074 | (1->3)-beta-D-glucan metabolic process | BIOLOGICAL_PROCESS | 0.00 | 0.00 |
| GO:0051171 | regulation of nitrogen compound metabolic process | BIOLOGICAL_PROCESS | 0.00 | 0.00 |
| GO:0045229 | external encapsulating structure organization | BIOLOGICAL_PROCESS | 0.00 | 0.00 |
| GO:0005740 | mitochondrial envelope | CELLULAR_COMPONENT | 0.00 | 0.00 |
| GO:0034470 | ncRNA processing | BIOLOGICAL_PROCESS | 0.00 | 0.00 |
| GO:0071467 | cellular response to pH | BIOLOGICAL_PROCESS | 0.00 | 0.00 |
| GO:0030004 | cellular monovalent inorganic cation homeostasis | BIOLOGICAL_PROCESS | 0.00 | 0.00 |
| GO:0080090 | regulation of primary metabolic process | BIOLOGICAL_PROCESS | 0.00 | 0.00 |
| GO:0040007 | growth | BIOLOGICAL_PROCESS | 0.00 | 0.00 |
| GO:0005816 | spindle pole body | CELLULAR_COMPONENT | 0.00 | 0.00 |
| GO:0043248 | proteasome assembly | BIOLOGICAL_PROCESS | 0.00 | 0.00 |
| GO:0032515 | negative regulation of phosphoprotein phosphatase activity | BIOLOGICAL_PROCESS | 0.00 | 0.00 |
| GO:0010923 | negative regulation of phosphatase activity | BIOLOGICAL_PROCESS | 0.00 | 0.00 |
| GO:0006403 | RNA localization | BIOLOGICAL_PROCESS | 0.00 | 0.00 |
| GO:0009607 | response to biotic stimulus | BIOLOGICAL_PROCESS | 0.00 | 0.00 |
| GO:0006605 | protein targeting | BIOLOGICAL_PROCESS | 0.00 | 0.00 |
| GO:0043467 | regulation of generation of precursor metabolites and energy | BIOLOGICAL_PROCESS | 0.00 | 0.00 |
| GO:0032993 | protein-DNA complex | CELLULAR_COMPONENT | 0.00 | 0.00 |
| GO:0000463 | maturation of LSU-rRNA from tricistronic rRNA transcript (SSU-rRNA, 5.8S rRNA, LSU-rRNA) | BIOLOGICAL_PROCESS | 0.00 | 0.00 |
| GO:0070682 | proteasome regulatory particle assembly | BIOLOGICAL_PROCESS | 0.00 | 0.00 |
| GO:0048878 | chemical homeostasis | BIOLOGICAL_PROCESS | 0.00 | 0.00 |
| GO:0022836 | gated channel activity | MOLECULAR_FUNCTION | 0.00 | 0.00 |
| GO:0016021 | integral component of membrane | CELLULAR_COMPONENT | 0.00 | 0.00 |
| GO:0036477 | somatodendritic compartment | CELLULAR_COMPONENT | 0.00 | 0.00 |
| GO:0071805 | potassium ion transmembrane transport | BIOLOGICAL_PROCESS | 0.00 | 0.00 |
| GO:0030594 | neurotransmitter receptor activity | MOLECULAR_FUNCTION | 0.00 | 0.00 |
| GO:0015079 | potassium ion transmembrane transporter activity | MOLECULAR_FUNCTION | 0.00 | 0.00 |
| GO:0016301 | kinase activity | MOLECULAR_FUNCTION | 0.00 | 0.00 |
| GO:0046824 | positive regulation of nucleocytoplasmic transport | BIOLOGICAL_PROCESS | 0.00 | 0.00 |
| GO:0051168 | nuclear export | BIOLOGICAL_PROCESS | 0.00 | 0.00 |
| GO:0016772 | transferase activity, transferring phosphorus-containing groups | MOLECULAR_FUNCTION | 0.00 | 0.00 |
| GO:0052547 | regulation of peptidase activity | BIOLOGICAL_PROCESS | 0.00 | 0.00 |
| GO:0070181 | small ribosomal subunit rRNA binding | MOLECULAR_FUNCTION | 0.00 | 0.00 |
| GO:0034727 | piecemeal microautophagy of the nucleus | BIOLOGICAL_PROCESS | 0.00 | 0.00 |
| GO:0090316 | positive regulation of intracellular protein transport | BIOLOGICAL_PROCESS | 0.00 | 0.00 |
| GO:0006405 | RNA export from nucleus | BIOLOGICAL_PROCESS | 0.00 | 0.00 |
| GO:0000451 | rRNA 2'-O-methylation | BIOLOGICAL_PROCESS | 0.00 | 0.00 |
| GO:0050658 | RNA transport | BIOLOGICAL_PROCESS | 0.00 | 0.00 |
| GO:0050657 | nucleic acid transport | BIOLOGICAL_PROCESS | 0.00 | 0.00 |
| GO:0051236 | establishment of RNA localization | BIOLOGICAL_PROCESS | 0.00 | 0.00 |
| GO:0006413 | translational initiation | BIOLOGICAL_PROCESS | 0.00 | 0.00 |
| GO:0009132 | nucleoside diphosphate metabolic process | BIOLOGICAL_PROCESS | 0.00 | 0.00 |
| GO:0036180 | filamentous growth of a population of unicellular organisms in response to biotic stimulus | BIOLOGICAL_PROCESS | 0.00 | 0.00 |
| GO:0005743 | mitochondrial inner membrane | CELLULAR_COMPONENT | 0.00 | 0.00 |
| GO:0006338 | chromatin remodeling | BIOLOGICAL_PROCESS | 0.00 | 0.00 |
| GO:0004865 | protein serine/threonine phosphatase inhibitor activity | MOLECULAR_FUNCTION | 0.00 | 0.00 |
| GO:0035305 | negative regulation of dephosphorylation | BIOLOGICAL_PROCESS | 0.00 | 0.00 |
| GO:0035308 | negative regulation of protein dephosphorylation | BIOLOGICAL_PROCESS | 0.00 | 0.00 |
| GO:0098781 | ncRNA transcription | BIOLOGICAL_PROCESS | 0.00 | 0.00 |
| GO:0050801 | ion homeostasis | BIOLOGICAL_PROCESS | 0.00 | 0.00 |
| GO:0042391 | regulation of membrane potential | BIOLOGICAL_PROCESS | 0.00 | 0.00 |
| GO:0031012 | extracellular matrix | CELLULAR_COMPONENT | 0.00 | 0.00 |
| GO:0098796 | membrane protein complex | CELLULAR_COMPONENT | 0.00 | 0.00 |
| GO:0034515 | proteasome storage granule | CELLULAR_COMPONENT | 0.00 | 0.00 |
| GO:0097064 | ncRNA export from nucleus | BIOLOGICAL_PROCESS | 0.00 | 0.00 |
| GO:0090605 | submerged biofilm formation | BIOLOGICAL_PROCESS | 0.00 | 0.00 |
| GO:0030864 | cortical actin cytoskeleton | CELLULAR_COMPONENT | 0.00 | 0.00 |
| GO:0005085 | guanyl-nucleotide exchange factor activity | MOLECULAR_FUNCTION | 0.00 | 0.00 |
| GO:0005758 | mitochondrial intermembrane space | CELLULAR_COMPONENT | 0.00 | 0.00 |
| GO:0031970 | organelle envelope lumen | CELLULAR_COMPONENT | 0.00 | 0.00 |
| GO:0003729 | mRNA binding | MOLECULAR_FUNCTION | 0.00 | 0.00 |
| GO:0031462 | Cul2-RING ubiquitin ligase complex | CELLULAR_COMPONENT | 0.00 | 0.00 |
| GO:0004474 | malate synthase activity | MOLECULAR_FUNCTION | 0.00 | 0.00 |
| GO:0006414 | translational elongation | BIOLOGICAL_PROCESS | 0.00 | 0.00 |
| GO:0034062 | 5'-3' RNA polymerase activity | MOLECULAR_FUNCTION | 0.00 | 0.00 |
| GO:0097747 | RNA polymerase activity | MOLECULAR_FUNCTION | 0.00 | 0.00 |
| GO:0031967 | organelle envelope | CELLULAR_COMPONENT | 0.00 | 0.00 |
| GO:0031975 | envelope | CELLULAR_COMPONENT | 0.00 | 0.00 |
| GO:0000479 | endonucleolytic cleavage of tricistronic rRNA transcript (SSU-rRNA, 5.8S rRNA, LSU-rRNA) | BIOLOGICAL_PROCESS | 0.00 | 0.00 |
| GO:0000478 | endonucleolytic cleavage involved in rRNA processing | BIOLOGICAL_PROCESS | 0.00 | 0.00 |
| GO:0071276 | cellular response to cadmium ion | BIOLOGICAL_PROCESS | 0.00 | 0.00 |
| GO:0033648 | host intracellular membrane-bounded organelle | CELLULAR_COMPONENT | 0.00 | 0.00 |
| GO:0033647 | host intracellular organelle | CELLULAR_COMPONENT | 0.00 | 0.00 |
| GO:0016477 | cell migration | BIOLOGICAL_PROCESS | 0.00 | 0.00 |
| GO:0042790 | nucleolar large rRNA transcription by RNA polymerase I | BIOLOGICAL_PROCESS | 0.00 | 0.00 |
| GO:0051278 | fungal-type cell wall polysaccharide biosynthetic process | BIOLOGICAL_PROCESS | 0.00 | 0.00 |
| GO:0007076 | mitotic chromosome condensation | BIOLOGICAL_PROCESS | 0.00 | 0.00 |
| GO:0046822 | regulation of nucleocytoplasmic transport | BIOLOGICAL_PROCESS | 0.00 | 0.00 |
| GO:0060589 | nucleoside-triphosphatase regulator activity | MOLECULAR_FUNCTION | 0.00 | 0.00 |
| GO:0030695 | GTPase regulator activity | MOLECULAR_FUNCTION | 0.00 | 0.00 |
| GO:0019438 | aromatic compound biosynthetic process | BIOLOGICAL_PROCESS | 0.00 | 0.00 |
| GO:0004712 | protein serine/threonine/tyrosine kinase activity | MOLECULAR_FUNCTION | 0.00 | 0.00 |
| GO:0031324 | negative regulation of cellular metabolic process | BIOLOGICAL_PROCESS | 0.00 | 0.00 |
| GO:0016070 | RNA metabolic process | BIOLOGICAL_PROCESS | 0.00 | 0.00 |
| GO:0001816 | cytokine production | BIOLOGICAL_PROCESS | 0.00 | 0.00 |
| GO:0001817 | regulation of cytokine production | BIOLOGICAL_PROCESS | 0.00 | 0.00 |
| GO:0000747 | conjugation with cellular fusion | BIOLOGICAL_PROCESS | 0.00 | 0.00 |
| GO:0019866 | organelle inner membrane | CELLULAR_COMPONENT | 0.00 | 0.00 |
| GO:0008061 | chitin binding | MOLECULAR_FUNCTION | 0.00 | 0.00 |
| GO:0048870 | cell motility | BIOLOGICAL_PROCESS | 0.00 | 0.00 |
| GO:0055086 | nucleobase-containing small molecule metabolic process | BIOLOGICAL_PROCESS | 0.00 | 0.00 |
| GO:0031323 | regulation of cellular metabolic process | BIOLOGICAL_PROCESS | 0.00 | 0.00 |
| GO:0000287 | magnesium ion binding | MOLECULAR_FUNCTION | 0.00 | 0.00 |
| GO:0015276 | ligand-gated ion channel activity | MOLECULAR_FUNCTION | 0.00 | 0.00 |
| GO:0022834 | ligand-gated channel activity | MOLECULAR_FUNCTION | 0.00 | 0.00 |
| GO:0006563 | L-serine metabolic process | BIOLOGICAL_PROCESS | 0.00 | 0.00 |
| GO:1900182 | positive regulation of protein localization to nucleus | BIOLOGICAL_PROCESS | 0.00 | 0.00 |
| GO:1902570 | protein localization to nucleolus | BIOLOGICAL_PROCESS | 0.00 | 0.00 |
| GO:0000452 | snoRNA guided rRNA 2'-O-methylation | BIOLOGICAL_PROCESS | 0.00 | 0.00 |
| GO:0017102 | methionyl glutamyl tRNA synthetase complex | CELLULAR_COMPONENT | 0.00 | 0.00 |
| GO:0070941 | eisosome assembly | BIOLOGICAL_PROCESS | 0.00 | 0.00 |
| GO:0072671 | mitochondria-associated ubiquitin-dependent protein catabolic process | BIOLOGICAL_PROCESS | 0.00 | 0.00 |
| GO:1990145 | maintenance of translational fidelity | BIOLOGICAL_PROCESS | 0.00 | 0.00 |
| GO:0034411 | cell wall (1->3)-beta-D-glucan biosynthetic process | BIOLOGICAL_PROCESS | 0.00 | 0.00 |
| GO:0034407 | cell wall (1->3)-beta-D-glucan metabolic process | BIOLOGICAL_PROCESS | 0.00 | 0.00 |
| GO:0071970 | fungal-type cell wall (1->3)-beta-D-glucan biosynthetic process | BIOLOGICAL_PROCESS | 0.00 | 0.00 |
| GO:0071969 | fungal-type cell wall (1->3)-beta-D-glucan metabolic process | BIOLOGICAL_PROCESS | 0.00 | 0.00 |
| GO:0016051 | carbohydrate biosynthetic process | BIOLOGICAL_PROCESS | 0.00 | 0.00 |
| GO:0071966 | fungal-type cell wall polysaccharide metabolic process | BIOLOGICAL_PROCESS | 0.00 | 0.00 |
| GO:0004451 | isocitrate lyase activity | MOLECULAR_FUNCTION | 0.00 | 0.00 |
| GO:0006366 | transcription by RNA polymerase II | BIOLOGICAL_PROCESS | 0.00 | 0.00 |
| GO:0003743 | translation initiation factor activity | MOLECULAR_FUNCTION | 0.00 | 0.00 |
| GO:0006886 | intracellular protein transport | BIOLOGICAL_PROCESS | 0.00 | 0.00 |
| GO:0040034 | regulation of development, heterochronic | BIOLOGICAL_PROCESS | 0.00 | 0.00 |
| GO:0007010 | cytoskeleton organization | BIOLOGICAL_PROCESS | 0.00 | 0.00 |
| GO:0098630 | aggregation of unicellular organisms | BIOLOGICAL_PROCESS | 0.00 | 0.00 |
| GO:0042710 | biofilm formation | BIOLOGICAL_PROCESS | 0.00 | 0.00 |
| GO:0055080 | cation homeostasis | BIOLOGICAL_PROCESS | 0.00 | 0.00 |
| GO:0071214 | cellular response to abiotic stimulus | BIOLOGICAL_PROCESS | 0.00 | 0.00 |
| GO:0104004 | cellular response to environmental stimulus | BIOLOGICAL_PROCESS | 0.00 | 0.00 |
| GO:0031224 | intrinsic component of membrane | CELLULAR_COMPONENT | 0.00 | 0.00 |
| GO:0044804 | autophagy of nucleus | BIOLOGICAL_PROCESS | 0.00 | 0.00 |
| GO:0006354 | DNA-templated transcription, elongation | BIOLOGICAL_PROCESS | 0.00 | 0.00 |
| GO:0007610 | behavior | BIOLOGICAL_PROCESS | 0.00 | 0.00 |
| GO:0098743 | cell aggregation | BIOLOGICAL_PROCESS | 0.00 | 0.00 |
| GO:0006942 | regulation of striated muscle contraction | BIOLOGICAL_PROCESS | 0.00 | 0.00 |
| GO:0044419 | biological process involved in interspecies interaction between organisms | BIOLOGICAL_PROCESS | 0.00 | 0.00 |
| GO:0040018 | positive regulation of multicellular organism growth | BIOLOGICAL_PROCESS | 0.00 | 0.00 |
| GO:0019693 | ribose phosphate metabolic process | BIOLOGICAL_PROCESS | 0.00 | 0.00 |
| GO:0046907 | intracellular transport | BIOLOGICAL_PROCESS | 0.00 | 0.00 |
| GO:0042307 | positive regulation of protein import into nucleus | BIOLOGICAL_PROCESS | 0.00 | 0.00 |
| GO:1901992 | positive regulation of mitotic cell cycle phase transition | BIOLOGICAL_PROCESS | 0.00 | 0.00 |
| GO:1903829 | positive regulation of protein localization | BIOLOGICAL_PROCESS | 0.00 | 0.00 |
| GO:0009066 | aspartate family amino acid metabolic process | BIOLOGICAL_PROCESS | 0.00 | 0.00 |
| GO:0009205 | purine ribonucleoside triphosphate metabolic process | BIOLOGICAL_PROCESS | 0.00 | 0.00 |
| GO:0006734 | NADH metabolic process | BIOLOGICAL_PROCESS | 0.00 | 0.00 |
| GO:0003899 | DNA-directed 5'-3' RNA polymerase activity | MOLECULAR_FUNCTION | 0.00 | 0.00 |
| GO:0031966 | mitochondrial membrane | CELLULAR_COMPONENT | 0.00 | 0.00 |
| GO:0072521 | purine-containing compound metabolic process | BIOLOGICAL_PROCESS | 0.00 | 0.00 |
| GO:0009069 | serine family amino acid metabolic process | BIOLOGICAL_PROCESS | 0.00 | 0.00 |
| GO:0009144 | purine nucleoside triphosphate metabolic process | BIOLOGICAL_PROCESS | 0.00 | 0.00 |
| GO:0018130 | heterocycle biosynthetic process | BIOLOGICAL_PROCESS | 0.00 | 0.00 |
| GO:0000469 | cleavage involved in rRNA processing | BIOLOGICAL_PROCESS | 0.00 | 0.00 |
| GO:0009070 | serine family amino acid biosynthetic process | BIOLOGICAL_PROCESS | 0.00 | 0.00 |
| GO:0006941 | striated muscle contraction | BIOLOGICAL_PROCESS | 0.00 | 0.00 |
| GO:0006090 | pyruvate metabolic process | BIOLOGICAL_PROCESS | 0.00 | 0.00 |
| GO:0061014 | positive regulation of mRNA catabolic process | BIOLOGICAL_PROCESS | 0.00 | 0.00 |
| GO:0046983 | protein dimerization activity | MOLECULAR_FUNCTION | 0.00 | 0.00 |
| GO:1903008 | organelle disassembly | BIOLOGICAL_PROCESS | 0.00 | 0.00 |
| GO:1903313 | positive regulation of mRNA metabolic process | BIOLOGICAL_PROCESS | 0.00 | 0.00 |
| GO:0006541 | glutamine metabolic process | BIOLOGICAL_PROCESS | 0.00 | 0.00 |
| GO:0009259 | ribonucleotide metabolic process | BIOLOGICAL_PROCESS | 0.00 | 0.00 |
| GO:0036177 | filamentous growth of a population of unicellular organisms in response to pH | BIOLOGICAL_PROCESS | 0.00 | 0.00 |
| GO:0009199 | ribonucleoside triphosphate metabolic process | BIOLOGICAL_PROCESS | 0.00 | 0.00 |
| GO:0099080 | supramolecular complex | CELLULAR_COMPONENT | 0.00 | 0.00 |
| GO:0033157 | regulation of intracellular protein transport | BIOLOGICAL_PROCESS | 0.00 | 0.00 |
| GO:0005267 | potassium channel activity | MOLECULAR_FUNCTION | 0.00 | 0.00 |
| GO:0009150 | purine ribonucleotide metabolic process | BIOLOGICAL_PROCESS | 0.00 | 0.00 |
| GO:0006913 | nucleocytoplasmic transport | BIOLOGICAL_PROCESS | 0.00 | 0.00 |
| GO:0010468 | regulation of gene expression | BIOLOGICAL_PROCESS | 0.00 | 0.00 |
| GO:0043656 | host intracellular region | CELLULAR_COMPONENT | 0.00 | 0.00 |
| GO:0010822 | positive regulation of mitochondrion organization | BIOLOGICAL_PROCESS | 0.00 | 0.00 |
| GO:0033646 | host intracellular part | CELLULAR_COMPONENT | 0.00 | 0.00 |
| GO:0051169 | nuclear transport | BIOLOGICAL_PROCESS | 0.00 | 0.00 |
| GO:0090609 | single-species submerged biofilm formation | BIOLOGICAL_PROCESS | 0.00 | 0.00 |
| GO:0098800 | inner mitochondrial membrane protein complex | CELLULAR_COMPONENT | 0.00 | 0.00 |
| GO:0051222 | positive regulation of protein transport | BIOLOGICAL_PROCESS | 0.00 | 0.00 |
| GO:0034654 | nucleobase-containing compound biosynthetic process | BIOLOGICAL_PROCESS | 0.00 | 0.00 |
| GO:0009185 | ribonucleoside diphosphate metabolic process | BIOLOGICAL_PROCESS | 0.00 | 0.00 |
| GO:0050881 | musculoskeletal movement | BIOLOGICAL_PROCESS | 0.00 | 0.00 |
| GO:0014819 | regulation of skeletal muscle contraction | BIOLOGICAL_PROCESS | 0.00 | 0.00 |
| GO:0043891 | glyceraldehyde-3-phosphate dehydrogenase (NAD(P)+) (phosphorylating) activity | MOLECULAR_FUNCTION | 0.00 | 0.00 |
| GO:0050905 | neuromuscular process | BIOLOGICAL_PROCESS | 0.00 | 0.00 |
| GO:0003009 | skeletal muscle contraction | BIOLOGICAL_PROCESS | 0.00 | 0.00 |
| GO:0004365 | glyceraldehyde-3-phosphate dehydrogenase (NAD+) (phosphorylating) activity | MOLECULAR_FUNCTION | 0.00 | 0.00 |
| GO:1905552 | positive regulation of protein localization to endoplasmic reticulum | BIOLOGICAL_PROCESS | 0.00 | 0.00 |
| GO:0090736 | MATH domain binding | MOLECULAR_FUNCTION | 0.00 | 0.00 |
| GO:0014722 | regulation of skeletal muscle contraction by calcium ion signaling | BIOLOGICAL_PROCESS | 0.00 | 0.00 |
| GO:0033036 | macromolecule localization | BIOLOGICAL_PROCESS | 0.00 | 0.00 |
| GO:0015931 | nucleobase-containing compound transport | BIOLOGICAL_PROCESS | 0.00 | 0.00 |
| GO:0050779 | RNA destabilization | BIOLOGICAL_PROCESS | 0.00 | 0.00 |
| GO:0061157 | mRNA destabilization | BIOLOGICAL_PROCESS | 0.00 | 0.00 |
| GO:0016052 | carbohydrate catabolic process | BIOLOGICAL_PROCESS | 0.00 | 0.00 |
| GO:0006353 | DNA-templated transcription, termination | BIOLOGICAL_PROCESS | 0.00 | 0.00 |
| GO:0019725 | cellular homeostasis | BIOLOGICAL_PROCESS | 0.00 | 0.00 |
| GO:0046034 | ATP metabolic process | BIOLOGICAL_PROCESS | 0.00 | 0.00 |
| GO:0003008 | system process | BIOLOGICAL_PROCESS | 0.00 | 0.00 |
| GO:0006163 | purine nucleotide metabolic process | BIOLOGICAL_PROCESS | 0.00 | 0.00 |
| GO:0005746 | mitochondrial respirasome | CELLULAR_COMPONENT | 0.00 | 0.00 |
| GO:0045254 | pyruvate dehydrogenase complex | CELLULAR_COMPONENT | 0.00 | 0.00 |
| GO:0031137 | regulation of conjugation with cellular fusion | BIOLOGICAL_PROCESS | 0.00 | 0.00 |
| GO:0034410 | cell wall beta-glucan biosynthetic process | BIOLOGICAL_PROCESS | 0.00 | 0.00 |
| GO:0034506 | chromosome, centromeric core domain | CELLULAR_COMPONENT | 0.00 | 0.00 |
| GO:0070880 | fungal-type cell wall beta-glucan biosynthetic process | BIOLOGICAL_PROCESS | 0.00 | 0.00 |
| GO:0016192 | vesicle-mediated transport | BIOLOGICAL_PROCESS | 0.00 | 0.00 |
| GO:0006407 | rRNA export from nucleus | BIOLOGICAL_PROCESS | 0.00 | 0.00 |
| GO:0043505 | CENP-A containing nucleosome | CELLULAR_COMPONENT | 0.00 | 0.00 |
| GO:0052031 | modulation by symbiont of host defense response | BIOLOGICAL_PROCESS | 0.00 | 0.00 |
| GO:0044003 | modulation by symbiont of host process | BIOLOGICAL_PROCESS | 0.00 | 0.00 |
| GO:0051029 | rRNA transport | BIOLOGICAL_PROCESS | 0.00 | 0.00 |
| GO:0044416 | induction by symbiont of host defense response | BIOLOGICAL_PROCESS | 0.00 | 0.00 |
| GO:0140455 | cytoplasm protein quality control | BIOLOGICAL_PROCESS | 0.00 | 0.00 |
| GO:0007005 | mitochondrion organization | BIOLOGICAL_PROCESS | 0.00 | 0.00 |
| GO:0005996 | monosaccharide metabolic process | BIOLOGICAL_PROCESS | 0.00 | 0.00 |
| GO:0016573 | histone acetylation | BIOLOGICAL_PROCESS | 0.00 | 0.00 |
| GO:0009268 | response to pH | BIOLOGICAL_PROCESS | 0.00 | 0.00 |
| GO:0031981 | nuclear lumen | CELLULAR_COMPONENT | 0.00 | 0.00 |
| GO:0018996 | molting cycle, collagen and cuticulin-based cuticle | BIOLOGICAL_PROCESS | 0.00 | 0.00 |
| GO:0016773 | phosphotransferase activity, alcohol group as acceptor | MOLECULAR_FUNCTION | 0.00 | 0.00 |
| GO:0015108 | chloride transmembrane transporter activity | MOLECULAR_FUNCTION | 0.00 | 0.00 |
| GO:0006839 | mitochondrial transport | BIOLOGICAL_PROCESS | 0.00 | 0.00 |
| GO:0006535 | cysteine biosynthetic process from serine | BIOLOGICAL_PROCESS | 0.00 | 0.00 |
| GO:0016833 | oxo-acid-lyase activity | MOLECULAR_FUNCTION | 0.00 | 0.00 |
| GO:1905550 | regulation of protein localization to endoplasmic reticulum | BIOLOGICAL_PROCESS | 0.00 | 0.00 |
| GO:0006361 | transcription initiation from RNA polymerase I promoter | BIOLOGICAL_PROCESS | 0.00 | 0.00 |
| GO:0005230 | extracellular ligand-gated ion channel activity | MOLECULAR_FUNCTION | 0.00 | 0.00 |
| GO:0120025 | plasma membrane bounded cell projection | CELLULAR_COMPONENT | 0.00 | 0.00 |
| GO:0065007 | biological regulation | BIOLOGICAL_PROCESS | 0.00 | 0.00 |
| GO:0044010 | single-species biofilm formation | BIOLOGICAL_PROCESS | 0.00 | 0.00 |
| GO:0006753 | nucleoside phosphate metabolic process | BIOLOGICAL_PROCESS | 0.00 | 0.00 |
| GO:0042626 | ATPase-coupled transmembrane transporter activity | MOLECULAR_FUNCTION | 0.00 | 0.00 |
| GO:0000045 | autophagosome assembly | BIOLOGICAL_PROCESS | 0.00 | 0.00 |
| GO:0050877 | nervous system process | BIOLOGICAL_PROCESS | 0.00 | 0.00 |
| GO:0006475 | internal protein amino acid acetylation | BIOLOGICAL_PROCESS | 0.00 | 0.00 |
| GO:0018394 | peptidyl-lysine acetylation | BIOLOGICAL_PROCESS | 0.00 | 0.00 |
| GO:0018393 | internal peptidyl-lysine acetylation | BIOLOGICAL_PROCESS | 0.00 | 0.00 |
| GO:0010638 | positive regulation of organelle organization | BIOLOGICAL_PROCESS | 0.00 | 0.00 |
| GO:0010494 | cytoplasmic stress granule | CELLULAR_COMPONENT | 0.00 | 0.00 |
| GO:0031669 | cellular response to nutrient levels | BIOLOGICAL_PROCESS | 0.00 | 0.00 |
| GO:0097402 | neuroblast migration | BIOLOGICAL_PROCESS | 0.00 | 0.00 |
| GO:0043657 | host cell | CELLULAR_COMPONENT | 0.00 | 0.00 |
| GO:0018995 | host cellular component | CELLULAR_COMPONENT | 0.00 | 0.00 |
| GO:0046658 | anchored component of plasma membrane | CELLULAR_COMPONENT | 0.00 | 0.00 |
| GO:0045338 | farnesyl diphosphate metabolic process | BIOLOGICAL_PROCESS | 0.00 | 0.00 |
| GO:0034406 | cell wall beta-glucan metabolic process | BIOLOGICAL_PROCESS | 0.00 | 0.00 |
| GO:0035821 | modulation of process of another organism | BIOLOGICAL_PROCESS | 0.00 | 0.00 |
| GO:0031161 | phosphatidylinositol catabolic process | BIOLOGICAL_PROCESS | 0.00 | 0.00 |
| GO:0070879 | fungal-type cell wall beta-glucan metabolic process | BIOLOGICAL_PROCESS | 0.00 | 0.00 |
| GO:0033643 | host cell part | CELLULAR_COMPONENT | 0.00 | 0.00 |
| GO:0070972 | protein localization to endoplasmic reticulum | BIOLOGICAL_PROCESS | 0.00 | 0.00 |
| GO:0006006 | glucose metabolic process | BIOLOGICAL_PROCESS | 0.00 | 0.00 |
| GO:0043666 | regulation of phosphoprotein phosphatase activity | BIOLOGICAL_PROCESS | 0.00 | 0.00 |
| GO:0045454 | cell redox homeostasis | BIOLOGICAL_PROCESS | 0.00 | 0.00 |
| GO:0036170 | filamentous growth of a population of unicellular organisms in response to starvation | BIOLOGICAL_PROCESS | 0.00 | 0.00 |
| GO:0010921 | regulation of phosphatase activity | BIOLOGICAL_PROCESS | 0.00 | 0.00 |
| GO:1905037 | autophagosome organization | BIOLOGICAL_PROCESS | 0.00 | 0.00 |
| GO:0009141 | nucleoside triphosphate metabolic process | BIOLOGICAL_PROCESS | 0.00 | 0.00 |
| GO:0015267 | channel activity | MOLECULAR_FUNCTION | 0.00 | 0.00 |
| GO:0022803 | passive transmembrane transporter activity | MOLECULAR_FUNCTION | 0.00 | 0.00 |
| GO:0036178 | filamentous growth of a population of unicellular organisms in response to neutral pH | BIOLOGICAL_PROCESS | 0.00 | 0.00 |
| GO:0140535 | intracellular protein-containing complex | CELLULAR_COMPONENT | 0.00 | 0.00 |
| GO:0009117 | nucleotide metabolic process | BIOLOGICAL_PROCESS | 0.00 | 0.00 |
| GO:0016879 | ligase activity, forming carbon-nitrogen bonds | MOLECULAR_FUNCTION | 0.00 | 0.00 |
| GO:0044297 | cell body | CELLULAR_COMPONENT | 0.00 | 0.00 |
| GO:0006165 | nucleoside diphosphate phosphorylation | BIOLOGICAL_PROCESS | 0.00 | 0.00 |
| GO:0046939 | nucleotide phosphorylation | BIOLOGICAL_PROCESS | 0.00 | 0.00 |
| GO:1903311 | regulation of mRNA metabolic process | BIOLOGICAL_PROCESS | 0.00 | 0.00 |
| GO:0031668 | cellular response to extracellular stimulus | BIOLOGICAL_PROCESS | 0.00 | 0.00 |
| GO:0071496 | cellular response to external stimulus | BIOLOGICAL_PROCESS | 0.00 | 0.00 |
| GO:0009082 | branched-chain amino acid biosynthetic process | BIOLOGICAL_PROCESS | 0.00 | 0.00 |
| GO:0001054 | RNA polymerase I activity | MOLECULAR_FUNCTION | 0.00 | 0.00 |
| GO:0009064 | glutamine family amino acid metabolic process | BIOLOGICAL_PROCESS | 0.00 | 0.00 |
| GO:0045836 | positive regulation of meiotic nuclear division | BIOLOGICAL_PROCESS | 0.00 | 0.00 |
| GO:0098803 | respiratory chain complex | CELLULAR_COMPONENT | 0.00 | 0.00 |
| GO:0036176 | response to neutral pH | BIOLOGICAL_PROCESS | 0.00 | 0.00 |
| GO:0047100 | glyceraldehyde-3-phosphate dehydrogenase (NADP+) (phosphorylating) activity | MOLECULAR_FUNCTION | 0.00 | 0.00 |
| GO:0017050 | D-erythro-sphingosine kinase activity | MOLECULAR_FUNCTION | 0.00 | 0.00 |
| GO:1902808 | positive regulation of cell cycle G1/S phase transition | BIOLOGICAL_PROCESS | 0.00 | 0.00 |
| GO:1900087 | positive regulation of G1/S transition of mitotic cell cycle | BIOLOGICAL_PROCESS | 0.00 | 0.00 |
| GO:0009506 | plasmodesma | CELLULAR_COMPONENT | 0.00 | 0.00 |
| GO:0031134 | sister chromatid biorientation | BIOLOGICAL_PROCESS | 0.00 | 0.00 |
| GO:0031138 | negative regulation of conjugation with cellular fusion | BIOLOGICAL_PROCESS | 0.00 | 0.00 |
| GO:0031048 | heterochromatin assembly by small RNA | BIOLOGICAL_PROCESS | 0.00 | 0.00 |
| GO:0055044 | symplast | CELLULAR_COMPONENT | 0.00 | 0.00 |
| GO:0030983 | mismatched DNA binding | MOLECULAR_FUNCTION | 0.00 | 0.00 |
| GO:1904951 | positive regulation of establishment of protein localization | BIOLOGICAL_PROCESS | 0.00 | 0.00 |
| GO:0090068 | positive regulation of cell cycle process | BIOLOGICAL_PROCESS | 0.00 | 0.00 |
| GO:0031011 | Ino80 complex | CELLULAR_COMPONENT | 0.00 | 0.00 |
| GO:0000054 | ribosomal subunit export from nucleus | BIOLOGICAL_PROCESS | 0.00 | 0.00 |
| GO:0033750 | ribosome localization | BIOLOGICAL_PROCESS | 0.00 | 0.00 |
| GO:0042303 | molting cycle | BIOLOGICAL_PROCESS | 0.00 | 0.00 |
| GO:0015031 | protein transport | BIOLOGICAL_PROCESS | 0.00 | 0.00 |
| GO:0009267 | cellular response to starvation | BIOLOGICAL_PROCESS | 0.00 | 0.00 |
| GO:0030437 | ascospore formation | BIOLOGICAL_PROCESS | 0.00 | 0.00 |
| GO:0045184 | establishment of protein localization | BIOLOGICAL_PROCESS | 0.00 | 0.00 |
| GO:0005934 | cellular bud tip | CELLULAR_COMPONENT | 0.00 | 0.00 |
| GO:0019674 | NAD metabolic process | BIOLOGICAL_PROCESS | 0.00 | 0.00 |
| GO:0019344 | cysteine biosynthetic process | BIOLOGICAL_PROCESS | 0.00 | 0.00 |
| GO:0007165 | signal transduction | BIOLOGICAL_PROCESS | 0.00 | 0.00 |
| GO:1902600 | proton transmembrane transport | BIOLOGICAL_PROCESS | 0.00 | 0.00 |
| GO:0051649 | establishment of localization in cell | BIOLOGICAL_PROCESS | 0.00 | 0.00 |
| GO:2000113 | negative regulation of cellular macromolecule biosynthetic process | BIOLOGICAL_PROCESS | 0.00 | 0.00 |
| GO:0023052 | signaling | BIOLOGICAL_PROCESS | 0.00 | 0.00 |
| GO:0046031 | ADP metabolic process | BIOLOGICAL_PROCESS | 0.00 | 0.00 |
| GO:1901135 | carbohydrate derivative metabolic process | BIOLOGICAL_PROCESS | 0.00 | 0.00 |
| GO:0001653 | peptide receptor activity | MOLECULAR_FUNCTION | 0.00 | 0.00 |
| GO:0006351 | transcription, DNA-templated | BIOLOGICAL_PROCESS | 0.00 | 0.00 |
| GO:0006821 | chloride transport | BIOLOGICAL_PROCESS | 0.00 | 0.00 |
| GO:0032774 | RNA biosynthetic process | BIOLOGICAL_PROCESS | 0.00 | 0.00 |
| GO:0005935 | cellular bud neck | CELLULAR_COMPONENT | 0.00 | 0.00 |
| GO:0051785 | positive regulation of nuclear division | BIOLOGICAL_PROCESS | 0.00 | 0.00 |
| GO:0043169 | cation binding | MOLECULAR_FUNCTION | 0.00 | 0.00 |
| GO:0070469 | respirasome | CELLULAR_COMPONENT | 0.00 | 0.00 |
| GO:0050793 | regulation of developmental process | BIOLOGICAL_PROCESS | 0.00 | 0.00 |
| GO:0050789 | regulation of biological process | BIOLOGICAL_PROCESS | 0.00 | 0.00 |
| GO:1900180 | regulation of protein localization to nucleus | BIOLOGICAL_PROCESS | 0.00 | 0.00 |
| GO:0030099 | myeloid cell differentiation | BIOLOGICAL_PROCESS | 0.00 | 0.00 |
| GO:0045899 | positive regulation of RNA polymerase II transcription preinitiation complex assembly | BIOLOGICAL_PROCESS | 0.00 | 0.00 |
| GO:0006626 | protein targeting to mitochondrion | BIOLOGICAL_PROCESS | 0.00 | 0.00 |
| GO:0043488 | regulation of mRNA stability | BIOLOGICAL_PROCESS | 0.00 | 0.00 |
| GO:0043487 | regulation of RNA stability | BIOLOGICAL_PROCESS | 0.00 | 0.00 |
| GO:0016798 | hydrolase activity, acting on glycosyl bonds | MOLECULAR_FUNCTION | 0.00 | 0.00 |
| GO:0097659 | nucleic acid-templated transcription | BIOLOGICAL_PROCESS | 0.00 | 0.00 |
| GO:0043005 | neuron projection | CELLULAR_COMPONENT | 0.00 | 0.00 |
| GO:0000494 | box C/D RNA 3'-end processing | BIOLOGICAL_PROCESS | 0.00 | 0.00 |
| GO:0006735 | NADH regeneration | BIOLOGICAL_PROCESS | 0.00 | 0.00 |
| GO:0042797 | tRNA transcription by RNA polymerase III | BIOLOGICAL_PROCESS | 0.00 | 0.00 |
| GO:0009304 | tRNA transcription | BIOLOGICAL_PROCESS | 0.00 | 0.00 |
| GO:0033967 | box C/D RNA metabolic process | BIOLOGICAL_PROCESS | 0.00 | 0.00 |
| GO:0034963 | box C/D RNA processing | BIOLOGICAL_PROCESS | 0.00 | 0.00 |
| GO:2000765 | regulation of cytoplasmic translation | BIOLOGICAL_PROCESS | 0.00 | 0.00 |
| GO:0000480 | endonucleolytic cleavage in 5'-ETS of tricistronic rRNA transcript (SSU-rRNA, 5.8S rRNA, LSU-rRNA) | BIOLOGICAL_PROCESS | 0.00 | 0.00 |
| GO:0046873 | metal ion transmembrane transporter activity | MOLECULAR_FUNCTION | 0.00 | 0.00 |
| GO:0061013 | regulation of mRNA catabolic process | BIOLOGICAL_PROCESS | 0.00 | 0.00 |
| GO:0060187 | cell pole | CELLULAR_COMPONENT | 0.00 | 0.00 |
| GO:0071944 | cell periphery | CELLULAR_COMPONENT | 0.00 | 0.00 |
| GO:0042995 | cell projection | CELLULAR_COMPONENT | 0.00 | 0.00 |
| GO:0051641 | cellular localization | BIOLOGICAL_PROCESS | 0.00 | 0.00 |
| GO:0006468 | protein phosphorylation | BIOLOGICAL_PROCESS | 0.00 | 0.00 |
| GO:0065002 | intracellular protein transmembrane transport | BIOLOGICAL_PROCESS | 0.00 | 0.00 |
| GO:0000819 | sister chromatid segregation | BIOLOGICAL_PROCESS | 0.00 | 0.00 |
| GO:0001731 | formation of translation preinitiation complex | BIOLOGICAL_PROCESS | 0.00 | 0.00 |
| GO:0050795 | regulation of behavior | BIOLOGICAL_PROCESS | 0.00 | 0.00 |
| GO:0016874 | ligase activity | MOLECULAR_FUNCTION | 0.00 | 0.00 |
| GO:0043531 | ADP binding | MOLECULAR_FUNCTION | 0.00 | 0.00 |
| GO:0045898 | regulation of RNA polymerase II transcription preinitiation complex assembly | BIOLOGICAL_PROCESS | 0.00 | 0.00 |
| GO:0051489 | regulation of filopodium assembly | BIOLOGICAL_PROCESS | 0.00 | 0.00 |
| GO:0006757 | ATP generation from ADP | BIOLOGICAL_PROCESS | 0.00 | 0.00 |
| GO:0015629 | actin cytoskeleton | CELLULAR_COMPONENT | 0.00 | 0.00 |
| GO:0006096 | glycolytic process | BIOLOGICAL_PROCESS | 0.00 | 0.00 |
| GO:0009135 | purine nucleoside diphosphate metabolic process | BIOLOGICAL_PROCESS | 0.00 | 0.00 |
| GO:0009179 | purine ribonucleoside diphosphate metabolic process | BIOLOGICAL_PROCESS | 0.00 | 0.00 |
| GO:0006368 | transcription elongation from RNA polymerase II promoter | BIOLOGICAL_PROCESS | 0.00 | 0.00 |
| GO:0005319 | lipid transporter activity | MOLECULAR_FUNCTION | 0.00 | 0.00 |
| GO:0004864 | protein phosphatase inhibitor activity | MOLECULAR_FUNCTION | 0.00 | 0.00 |
| GO:0019212 | phosphatase inhibitor activity | MOLECULAR_FUNCTION | 0.00 | 0.00 |
| GO:1990542 | mitochondrial transmembrane transport | BIOLOGICAL_PROCESS | 0.00 | 0.00 |
| GO:1903047 | mitotic cell cycle process | BIOLOGICAL_PROCESS | 0.00 | 0.00 |
| GO:0017148 | negative regulation of translation | BIOLOGICAL_PROCESS | 0.00 | 0.00 |
| GO:0015078 | proton transmembrane transporter activity | MOLECULAR_FUNCTION | 0.00 | 0.00 |
| GO:0045927 | positive regulation of growth | BIOLOGICAL_PROCESS | 0.00 | 0.00 |
| GO:0071806 | protein transmembrane transport | BIOLOGICAL_PROCESS | 0.00 | 0.00 |
| GO:0006298 | mismatch repair | BIOLOGICAL_PROCESS | 0.00 | 0.00 |
| GO:0042594 | response to starvation | BIOLOGICAL_PROCESS | 0.00 | 0.00 |
| GO:0031325 | positive regulation of cellular metabolic process | BIOLOGICAL_PROCESS | 0.00 | 0.00 |
| GO:0019637 | organophosphate metabolic process | BIOLOGICAL_PROCESS | 0.00 | 0.00 |
| GO:0040012 | regulation of locomotion | BIOLOGICAL_PROCESS | 0.00 | 0.00 |
| GO:0071705 | nitrogen compound transport | BIOLOGICAL_PROCESS | 0.00 | 0.00 |
| GO:0043025 | neuronal cell body | CELLULAR_COMPONENT | 0.00 | 0.00 |
| GO:0019898 | extrinsic component of membrane | CELLULAR_COMPONENT | 0.00 | 0.00 |
| GO:0097447 | dendritic tree | CELLULAR_COMPONENT | 0.00 | 0.00 |
| GO:0030425 | dendrite | CELLULAR_COMPONENT | 0.00 | 0.00 |
| GO:0042306 | regulation of protein import into nucleus | BIOLOGICAL_PROCESS | 0.00 | 0.00 |
| GO:0033179 | proton-transporting V-type ATPase, V0 domain | CELLULAR_COMPONENT | 0.00 | 0.00 |
| GO:0031139 | positive regulation of conjugation with cellular fusion | BIOLOGICAL_PROCESS | 0.00 | 0.00 |
| GO:0006892 | post-Golgi vesicle-mediated transport | BIOLOGICAL_PROCESS | 0.00 | 0.00 |
| GO:0019318 | hexose metabolic process | BIOLOGICAL_PROCESS | 0.00 | 0.00 |
| GO:0046872 | metal ion binding | MOLECULAR_FUNCTION | 0.00 | 0.00 |
| GO:0006473 | protein acetylation | BIOLOGICAL_PROCESS | 0.00 | 0.00 |
| GO:0022900 | electron transport chain | BIOLOGICAL_PROCESS | 0.00 | 0.00 |
| GO:0006084 | acetyl-CoA metabolic process | BIOLOGICAL_PROCESS | 0.00 | 0.00 |
| GO:0015698 | inorganic anion transport | BIOLOGICAL_PROCESS | 0.00 | 0.00 |
| GO:1902407 | assembly of actomyosin apparatus involved in mitotic cytokinesis | BIOLOGICAL_PROCESS | 0.00 | 0.00 |
| GO:0034249 | negative regulation of cellular amide metabolic process | BIOLOGICAL_PROCESS | 0.00 | 0.00 |
| GO:0005976 | polysaccharide metabolic process | BIOLOGICAL_PROCESS | 0.00 | 0.00 |
| GO:1903475 | mitotic actomyosin contractile ring assembly | BIOLOGICAL_PROCESS | 0.00 | 0.00 |
| GO:0016787 | hydrolase activity | MOLECULAR_FUNCTION | 0.00 | 0.00 |
| GO:0140098 | catalytic activity, acting on RNA | MOLECULAR_FUNCTION | 0.00 | 0.00 |
| GO:0010563 | negative regulation of phosphorus metabolic process | BIOLOGICAL_PROCESS | 0.00 | 0.00 |
| GO:0045936 | negative regulation of phosphate metabolic process | BIOLOGICAL_PROCESS | 0.00 | 0.00 |
| GO:0010927 | cellular component assembly involved in morphogenesis | BIOLOGICAL_PROCESS | 0.00 | 0.00 |
| GO:0040011 | locomotion | BIOLOGICAL_PROCESS | 0.00 | 0.00 |
| GO:0005634 | nucleus | CELLULAR_COMPONENT | 0.01 | 0.00 |
| GO:0045933 | positive regulation of muscle contraction | BIOLOGICAL_PROCESS | 0.01 | 0.00 |
| GO:0031252 | cell leading edge | CELLULAR_COMPONENT | 0.01 | 0.00 |
| GO:0000329 | fungal-type vacuole membrane | CELLULAR_COMPONENT | 0.01 | 0.00 |
| GO:0000466 | maturation of 5.8S rRNA from tricistronic rRNA transcript (SSU-rRNA, 5.8S rRNA, LSU-rRNA) | BIOLOGICAL_PROCESS | 0.01 | 0.00 |
| GO:0006670 | sphingosine metabolic process | BIOLOGICAL_PROCESS | 0.01 | 0.00 |
| GO:0005665 | RNA polymerase II, core complex | CELLULAR_COMPONENT | 0.01 | 0.00 |
| GO:0016973 | poly(A)+ mRNA export from nucleus | BIOLOGICAL_PROCESS | 0.01 | 0.00 |
| GO:0046512 | sphingosine biosynthetic process | BIOLOGICAL_PROCESS | 0.01 | 0.00 |
| GO:0046520 | sphingoid biosynthetic process | BIOLOGICAL_PROCESS | 0.01 | 0.00 |
| GO:0052200 | response to host defenses | BIOLOGICAL_PROCESS | 0.01 | 0.00 |
| GO:0052173 | response to defenses of other organism | BIOLOGICAL_PROCESS | 0.01 | 0.00 |
| GO:0048639 | positive regulation of developmental growth | BIOLOGICAL_PROCESS | 0.01 | 0.00 |
| GO:0044087 | regulation of cellular component biogenesis | BIOLOGICAL_PROCESS | 0.01 | 0.00 |
| GO:0075136 | response to host | BIOLOGICAL_PROCESS | 0.01 | 0.00 |
| GO:0007548 | sex differentiation | BIOLOGICAL_PROCESS | 0.01 | 0.00 |
| GO:0007033 | vacuole organization | BIOLOGICAL_PROCESS | 0.01 | 0.00 |
| GO:0032984 | protein-containing complex disassembly | BIOLOGICAL_PROCESS | 0.01 | 0.00 |
| GO:0005777 | peroxisome | CELLULAR_COMPONENT | 0.01 | 0.00 |
| GO:0042579 | microbody | CELLULAR_COMPONENT | 0.01 | 0.00 |
| GO:0140014 | mitotic nuclear division | BIOLOGICAL_PROCESS | 0.01 | 0.00 |
| GO:0005253 | anion channel activity | MOLECULAR_FUNCTION | 0.01 | 0.00 |
| GO:0015081 | sodium ion transmembrane transporter activity | MOLECULAR_FUNCTION | 0.01 | 0.00 |
| GO:0036244 | cellular response to neutral pH | BIOLOGICAL_PROCESS | 0.01 | 0.00 |
| GO:0051123 | RNA polymerase II preinitiation complex assembly | BIOLOGICAL_PROCESS | 0.01 | 0.00 |
| GO:0004672 | protein kinase activity | MOLECULAR_FUNCTION | 0.01 | 0.00 |
| GO:0015103 | inorganic anion transmembrane transporter activity | MOLECULAR_FUNCTION | 0.01 | 0.00 |
| GO:0030261 | chromosome condensation | BIOLOGICAL_PROCESS | 0.01 | 0.00 |
| GO:0043648 | dicarboxylic acid metabolic process | BIOLOGICAL_PROCESS | 0.01 | 0.00 |
| GO:0000425 | pexophagy | BIOLOGICAL_PROCESS | 0.01 | 0.00 |
| GO:0000464 | endonucleolytic cleavage in ITS1 upstream of 5.8S rRNA from tricistronic rRNA transcript (SSU-rRNA, 5.8S rRNA, LSU-rRNA) | BIOLOGICAL_PROCESS | 0.01 | 0.00 |
| GO:0036286 | eisosome filament | CELLULAR_COMPONENT | 0.01 | 0.00 |
| GO:0031383 | regulation of mating projection assembly | BIOLOGICAL_PROCESS | 0.01 | 0.00 |
| GO:0017077 | oxidative phosphorylation uncoupler activity | MOLECULAR_FUNCTION | 0.01 | 0.00 |
| GO:1990813 | meiotic centromeric cohesion protection | BIOLOGICAL_PROCESS | 0.01 | 0.00 |
| GO:1990823 | response to leukemia inhibitory factor | BIOLOGICAL_PROCESS | 0.01 | 0.00 |
| GO:1990830 | cellular response to leukemia inhibitory factor | BIOLOGICAL_PROCESS | 0.01 | 0.00 |
| GO:1903599 | positive regulation of autophagy of mitochondrion | BIOLOGICAL_PROCESS | 0.01 | 0.00 |
| GO:0030183 | B cell differentiation | BIOLOGICAL_PROCESS | 0.01 | 0.00 |
| GO:0042113 | B cell activation | BIOLOGICAL_PROCESS | 0.01 | 0.00 |
| GO:0005757 | mitochondrial permeability transition pore complex | CELLULAR_COMPONENT | 0.01 | 0.00 |
| GO:1902930 | regulation of alcohol biosynthetic process | BIOLOGICAL_PROCESS | 0.01 | 0.00 |
| GO:1901526 | positive regulation of mitophagy | BIOLOGICAL_PROCESS | 0.01 | 0.00 |
| GO:0106118 | regulation of sterol biosynthetic process | BIOLOGICAL_PROCESS | 0.01 | 0.00 |
| GO:1902633 | 1-phosphatidyl-1D-myo-inositol 4,5-bisphosphate metabolic process | BIOLOGICAL_PROCESS | 0.01 | 0.00 |
| GO:0031982 | vesicle | CELLULAR_COMPONENT | 0.01 | 0.00 |
| GO:0007034 | vacuolar transport | BIOLOGICAL_PROCESS | 0.01 | 0.00 |
| GO:1901900 | regulation of protein localization to cell division site | BIOLOGICAL_PROCESS | 0.01 | 0.00 |
| GO:0005967 | mitochondrial pyruvate dehydrogenase complex | CELLULAR_COMPONENT | 0.01 | 0.00 |
| GO:0035875 | maintenance of meiotic sister chromatid cohesion, centromeric | BIOLOGICAL_PROCESS | 0.01 | 0.00 |
| GO:0034517 | ribophagy | BIOLOGICAL_PROCESS | 0.01 | 0.00 |
| GO:0048027 | mRNA 5'-UTR binding | MOLECULAR_FUNCTION | 0.01 | 0.00 |
| GO:0097010 | eukaryotic translation initiation factor 4F complex assembly | BIOLOGICAL_PROCESS | 0.01 | 0.00 |
| GO:0097002 | mitochondrial inner boundary membrane | CELLULAR_COMPONENT | 0.01 | 0.00 |
| GO:0032443 | regulation of ergosterol biosynthetic process | BIOLOGICAL_PROCESS | 0.01 | 0.00 |
| GO:0071817 | MMXD complex | CELLULAR_COMPONENT | 0.01 | 0.00 |
| GO:0055067 | monovalent inorganic cation homeostasis | BIOLOGICAL_PROCESS | 0.01 | 0.00 |
| GO:0003852 | 2-isopropylmalate synthase activity | MOLECULAR_FUNCTION | 0.01 | 0.00 |
| GO:0005736 | RNA polymerase I complex | CELLULAR_COMPONENT | 0.01 | 0.00 |
| GO:1990234 | transferase complex | CELLULAR_COMPONENT | 0.01 | 0.00 |
| GO:0005856 | cytoskeleton | CELLULAR_COMPONENT | 0.01 | 0.00 |
| GO:0045787 | positive regulation of cell cycle | BIOLOGICAL_PROCESS | 0.01 | 0.00 |
| GO:0016459 | myosin complex | CELLULAR_COMPONENT | 0.01 | 0.00 |
| GO:0009055 | electron transfer activity | MOLECULAR_FUNCTION | 0.01 | 0.00 |
| GO:0051082 | unfolded protein binding | MOLECULAR_FUNCTION | 0.01 | 0.00 |
| GO:1901989 | positive regulation of cell cycle phase transition | BIOLOGICAL_PROCESS | 0.01 | 0.00 |
| GO:0000288 | nuclear-transcribed mRNA catabolic process, deadenylation-dependent decay | BIOLOGICAL_PROCESS | 0.01 | 0.00 |
| GO:0022904 | respiratory electron transport chain | BIOLOGICAL_PROCESS | 0.01 | 0.00 |
| GO:0016765 | transferase activity, transferring alkyl or aryl (other than methyl) groups | MOLECULAR_FUNCTION | 0.01 | 0.00 |
| GO:0006637 | acyl-CoA metabolic process | BIOLOGICAL_PROCESS | 0.01 | 0.00 |
| GO:0035383 | thioester metabolic process | BIOLOGICAL_PROCESS | 0.01 | 0.00 |
| GO:0005216 | ion channel activity | MOLECULAR_FUNCTION | 0.01 | 0.00 |
| GO:0032787 | monocarboxylic acid metabolic process | BIOLOGICAL_PROCESS | 0.01 | 0.00 |
| GO:0031267 | small GTPase binding | MOLECULAR_FUNCTION | 0.01 | 0.00 |
| GO:0009081 | branched-chain amino acid metabolic process | BIOLOGICAL_PROCESS | 0.01 | 0.00 |
| GO:0015453 | oxidoreduction-driven active transmembrane transporter activity | MOLECULAR_FUNCTION | 0.01 | 0.00 |
| GO:0032506 | cytokinetic process | BIOLOGICAL_PROCESS | 0.01 | 0.00 |
| GO:0045935 | positive regulation of nucleobase-containing compound metabolic process | BIOLOGICAL_PROCESS | 0.01 | 0.00 |
| GO:0032153 | cell division site | CELLULAR_COMPONENT | 0.01 | 0.00 |
| GO:0031234 | extrinsic component of cytoplasmic side of plasma membrane | CELLULAR_COMPONENT | 0.01 | 0.00 |
| GO:0030241 | skeletal muscle myosin thick filament assembly | BIOLOGICAL_PROCESS | 0.01 | 0.00 |
| GO:0006417 | regulation of translation | BIOLOGICAL_PROCESS | 0.01 | 0.00 |
| GO:0097346 | INO80-type complex | CELLULAR_COMPONENT | 0.01 | 0.00 |
| GO:0000056 | ribosomal small subunit export from nucleus | BIOLOGICAL_PROCESS | 0.01 | 0.00 |
| GO:0034605 | cellular response to heat | BIOLOGICAL_PROCESS | 0.01 | 0.00 |
| GO:0048519 | negative regulation of biological process | BIOLOGICAL_PROCESS | 0.01 | 0.00 |
| GO:0006814 | sodium ion transport | BIOLOGICAL_PROCESS | 0.01 | 0.00 |
| GO:0015293 | symporter activity | MOLECULAR_FUNCTION | 0.01 | 0.00 |
| GO:0008528 | G protein-coupled peptide receptor activity | MOLECULAR_FUNCTION | 0.01 | 0.00 |
| GO:0044743 | protein transmembrane import into intracellular organelle | BIOLOGICAL_PROCESS | 0.01 | 0.00 |
| GO:0030001 | metal ion transport | BIOLOGICAL_PROCESS | 0.01 | 0.00 |
| GO:0016614 | oxidoreductase activity, acting on CH-OH group of donors | MOLECULAR_FUNCTION | 0.01 | 0.00 |
| GO:0010558 | negative regulation of macromolecule biosynthetic process | BIOLOGICAL_PROCESS | 0.01 | 0.00 |
| GO:0006097 | glyoxylate cycle | BIOLOGICAL_PROCESS | 0.01 | 0.00 |
| GO:0031032 | actomyosin structure organization | BIOLOGICAL_PROCESS | 0.01 | 0.00 |
| GO:0005254 | chloride channel activity | MOLECULAR_FUNCTION | 0.01 | 0.00 |
| GO:0090304 | nucleic acid metabolic process | BIOLOGICAL_PROCESS | 0.01 | 0.00 |
| GO:0072655 | establishment of protein localization to mitochondrion | BIOLOGICAL_PROCESS | 0.01 | 0.00 |
| GO:0070585 | protein localization to mitochondrion | BIOLOGICAL_PROCESS | 0.01 | 0.00 |
| GO:0048646 | anatomical structure formation involved in morphogenesis | BIOLOGICAL_PROCESS | 0.01 | 0.00 |
| GO:0000070 | mitotic sister chromatid segregation | BIOLOGICAL_PROCESS | 0.01 | 0.00 |
| GO:0060261 | positive regulation of transcription initiation from RNA polymerase II promoter | BIOLOGICAL_PROCESS | 0.01 | 0.00 |
| GO:0002253 | activation of immune response | BIOLOGICAL_PROCESS | 0.01 | 0.00 |
| GO:0034248 | regulation of cellular amide metabolic process | BIOLOGICAL_PROCESS | 0.01 | 0.00 |
| GO:0030866 | cortical actin cytoskeleton organization | BIOLOGICAL_PROCESS | 0.01 | 0.00 |
| GO:0033692 | cellular polysaccharide biosynthetic process | BIOLOGICAL_PROCESS | 0.01 | 0.00 |
| GO:0044011 | single-species biofilm formation on inanimate substrate | BIOLOGICAL_PROCESS | 0.01 | 0.00 |
| GO:0030139 | endocytic vesicle | CELLULAR_COMPONENT | 0.01 | 0.00 |
| GO:0031507 | heterochromatin assembly | BIOLOGICAL_PROCESS | 0.01 | 0.00 |
| GO:0016592 | mediator complex | CELLULAR_COMPONENT | 0.01 | 0.00 |
| GO:0051020 | GTPase binding | MOLECULAR_FUNCTION | 0.01 | 0.00 |
| GO:0051028 | mRNA transport | BIOLOGICAL_PROCESS | 0.01 | 0.00 |
| GO:0051223 | regulation of protein transport | BIOLOGICAL_PROCESS | 0.01 | 0.00 |
| GO:0061458 | reproductive system development | BIOLOGICAL_PROCESS | 0.01 | 0.00 |
| GO:0048608 | reproductive structure development | BIOLOGICAL_PROCESS | 0.01 | 0.00 |
| GO:0000271 | polysaccharide biosynthetic process | BIOLOGICAL_PROCESS | 0.01 | 0.00 |
| GO:0070828 | heterochromatin organization | BIOLOGICAL_PROCESS | 0.01 | 0.00 |
| GO:0050879 | multicellular organismal movement | BIOLOGICAL_PROCESS | 0.01 | 0.00 |
| GO:0060260 | regulation of transcription initiation from RNA polymerase II promoter | BIOLOGICAL_PROCESS | 0.01 | 0.00 |
| GO:0005200 | structural constituent of cytoskeleton | MOLECULAR_FUNCTION | 0.01 | 0.00 |
| GO:0008422 | beta-glucosidase activity | MOLECULAR_FUNCTION | 0.01 | 0.00 |
| GO:0110020 | regulation of actomyosin structure organization | BIOLOGICAL_PROCESS | 0.01 | 0.00 |
| GO:0070180 | large ribosomal subunit rRNA binding | MOLECULAR_FUNCTION | 0.01 | 0.00 |
| GO:0022413 | reproductive process in single-celled organism | BIOLOGICAL_PROCESS | 0.01 | 0.00 |
| GO:0006026 | aminoglycan catabolic process | BIOLOGICAL_PROCESS | 0.01 | 0.00 |
| GO:0008104 | protein localization | BIOLOGICAL_PROCESS | 0.01 | 0.00 |
| GO:0070727 | cellular macromolecule localization | BIOLOGICAL_PROCESS | 0.01 | 0.00 |
| GO:0016903 | oxidoreductase activity, acting on the aldehyde or oxo group of donors | MOLECULAR_FUNCTION | 0.01 | 0.00 |
| GO:0031497 | chromatin assembly | BIOLOGICAL_PROCESS | 0.01 | 0.00 |
| GO:0035303 | regulation of dephosphorylation | BIOLOGICAL_PROCESS | 0.01 | 0.00 |
| GO:0035304 | regulation of protein dephosphorylation | BIOLOGICAL_PROCESS | 0.01 | 0.00 |
| GO:0000278 | mitotic cell cycle | BIOLOGICAL_PROCESS | 0.01 | 0.00 |
| GO:0044264 | cellular polysaccharide metabolic process | BIOLOGICAL_PROCESS | 0.01 | 0.00 |
| GO:0044837 | actomyosin contractile ring organization | BIOLOGICAL_PROCESS | 0.01 | 0.00 |
| GO:0032505 | reproduction of a single-celled organism | BIOLOGICAL_PROCESS | 0.01 | 0.00 |
| GO:0008608 | attachment of spindle microtubules to kinetochore | BIOLOGICAL_PROCESS | 0.01 | 0.00 |
| GO:0031327 | negative regulation of cellular biosynthetic process | BIOLOGICAL_PROCESS | 0.01 | 0.00 |
| GO:0009678 | pyrophosphate hydrolysis-driven proton transmembrane transporter activity | MOLECULAR_FUNCTION | 0.01 | 0.00 |
| GO:0051130 | positive regulation of cellular component organization | BIOLOGICAL_PROCESS | 0.01 | 0.00 |
| GO:0071824 | protein-DNA complex subunit organization | BIOLOGICAL_PROCESS | 0.01 | 0.00 |
| GO:0006790 | sulfur compound metabolic process | BIOLOGICAL_PROCESS | 0.01 | 0.00 |
| GO:0051984 | positive regulation of chromosome segregation | BIOLOGICAL_PROCESS | 0.01 | 0.00 |
| GO:0090501 | RNA phosphodiester bond hydrolysis | BIOLOGICAL_PROCESS | 0.01 | 0.00 |
| GO:0030523 | dihydrolipoamide S-acyltransferase activity | MOLECULAR_FUNCTION | 0.01 | 0.00 |
| GO:0051219 | phosphoprotein binding | MOLECULAR_FUNCTION | 0.01 | 0.00 |
| GO:0045337 | farnesyl diphosphate biosynthetic process | BIOLOGICAL_PROCESS | 0.01 | 0.00 |
| GO:0001055 | RNA polymerase II activity | MOLECULAR_FUNCTION | 0.01 | 0.00 |
| GO:0051754 | meiotic sister chromatid cohesion, centromeric | BIOLOGICAL_PROCESS | 0.01 | 0.00 |
| GO:0000142 | cellular bud neck contractile ring | CELLULAR_COMPONENT | 0.01 | 0.00 |
| GO:0001172 | transcription, RNA-templated | BIOLOGICAL_PROCESS | 0.01 | 0.00 |
| GO:0070601 | centromeric sister chromatid cohesion | BIOLOGICAL_PROCESS | 0.01 | 0.00 |
| GO:0010608 | post-transcriptional regulation of gene expression | BIOLOGICAL_PROCESS | 0.01 | 0.00 |
| GO:2000144 | positive regulation of DNA-templated transcription, initiation | BIOLOGICAL_PROCESS | 0.01 | 0.00 |
| GO:0051286 | cell tip | CELLULAR_COMPONENT | 0.01 | 0.00 |
| GO:0009893 | positive regulation of metabolic process | BIOLOGICAL_PROCESS | 0.01 | 0.00 |
| GO:0055082 | cellular chemical homeostasis | BIOLOGICAL_PROCESS | 0.01 | 0.00 |
| GO:0051254 | positive regulation of RNA metabolic process | BIOLOGICAL_PROCESS | 0.01 | 0.00 |
| GO:0044272 | sulfur compound biosynthetic process | BIOLOGICAL_PROCESS | 0.01 | 0.00 |
| GO:0031334 | positive regulation of protein-containing complex assembly | BIOLOGICAL_PROCESS | 0.01 | 0.00 |
| GO:0048522 | positive regulation of cellular process | BIOLOGICAL_PROCESS | 0.01 | 0.00 |
| GO:0019829 | ATPase-coupled cation transmembrane transporter activity | MOLECULAR_FUNCTION | 0.01 | 0.00 |
| GO:0000502 | proteasome complex | CELLULAR_COMPONENT | 0.01 | 0.00 |
| GO:0031326 | regulation of cellular biosynthetic process | BIOLOGICAL_PROCESS | 0.01 | 0.00 |
| GO:0043543 | protein acylation | BIOLOGICAL_PROCESS | 0.01 | 0.00 |
| GO:0051302 | regulation of cell division | BIOLOGICAL_PROCESS | 0.01 | 0.00 |
| GO:0035267 | NuA4 histone acetyltransferase complex | CELLULAR_COMPONENT | 0.01 | 0.00 |
| GO:0014866 | skeletal myofibril assembly | BIOLOGICAL_PROCESS | 0.01 | 0.00 |
| GO:0000812 | Swr1 complex | CELLULAR_COMPONENT | 0.01 | 0.00 |
| GO:0051031 | tRNA transport | BIOLOGICAL_PROCESS | 0.01 | 0.00 |
| GO:0046475 | glycerophospholipid catabolic process | BIOLOGICAL_PROCESS | 0.01 | 0.00 |
| GO:0043189 | H4/H2A histone acetyltransferase complex | CELLULAR_COMPONENT | 0.01 | 0.00 |
| GO:1901990 | regulation of mitotic cell cycle phase transition | BIOLOGICAL_PROCESS | 0.01 | 0.00 |
| GO:0045931 | positive regulation of mitotic cell cycle | BIOLOGICAL_PROCESS | 0.01 | 0.00 |
| GO:0005929 | cilium | CELLULAR_COMPONENT | 0.02 | 0.00 |
| GO:0098687 | chromosomal region | CELLULAR_COMPONENT | 0.02 | 0.00 |
| GO:0030865 | cortical cytoskeleton organization | BIOLOGICAL_PROCESS | 0.02 | 0.00 |
| GO:0099512 | supramolecular fiber | CELLULAR_COMPONENT | 0.02 | 0.00 |
| GO:0036474 | cell death in response to hydrogen peroxide | BIOLOGICAL_PROCESS | 0.02 | 0.00 |
| GO:2000045 | regulation of G1/S transition of mitotic cell cycle | BIOLOGICAL_PROCESS | 0.02 | 0.00 |
| GO:0050810 | regulation of steroid biosynthetic process | BIOLOGICAL_PROCESS | 0.02 | 0.00 |
| GO:0006406 | mRNA export from nucleus | BIOLOGICAL_PROCESS | 0.02 | 0.00 |
| GO:0006452 | translational frameshifting | BIOLOGICAL_PROCESS | 0.02 | 0.00 |
| GO:0048285 | organelle fission | BIOLOGICAL_PROCESS | 0.02 | 0.00 |
| GO:0042025 | host cell nucleus | CELLULAR_COMPONENT | 0.02 | 0.00 |
| GO:0070911 | global genome nucleotide-excision repair | BIOLOGICAL_PROCESS | 0.02 | 0.00 |
| GO:0044772 | mitotic cell cycle phase transition | BIOLOGICAL_PROCESS | 0.02 | 0.00 |
| GO:0000935 | division septum | CELLULAR_COMPONENT | 0.02 | 0.00 |
| GO:0000915 | actomyosin contractile ring assembly | BIOLOGICAL_PROCESS | 0.02 | 0.00 |
| GO:0000912 | assembly of actomyosin apparatus involved in cytokinesis | BIOLOGICAL_PROCESS | 0.02 | 0.00 |
| GO:1901524 | regulation of mitophagy | BIOLOGICAL_PROCESS | 0.02 | 0.00 |
| GO:0072380 | TRC complex | CELLULAR_COMPONENT | 0.02 | 0.00 |
| GO:0006808 | regulation of nitrogen utilization | BIOLOGICAL_PROCESS | 0.02 | 0.00 |
| GO:0004338 | glucan exo-1,3-beta-glucosidase activity | MOLECULAR_FUNCTION | 0.02 | 0.00 |
| GO:0044094 | host cell nuclear part | CELLULAR_COMPONENT | 0.02 | 0.00 |
| GO:0019034 | viral replication complex | CELLULAR_COMPONENT | 0.02 | 0.00 |
| GO:0007089 | traversing start control point of mitotic cell cycle | BIOLOGICAL_PROCESS | 0.02 | 0.00 |
| GO:0071629 | cytoplasm protein quality control by the ubiquitin-proteasome system | BIOLOGICAL_PROCESS | 0.02 | 0.00 |
| GO:0009853 | photorespiration | BIOLOGICAL_PROCESS | 0.02 | 0.00 |
| GO:0110085 | mitotic actomyosin contractile ring | CELLULAR_COMPONENT | 0.02 | 0.00 |
| GO:0010591 | regulation of lamellipodium assembly | BIOLOGICAL_PROCESS | 0.02 | 0.00 |
| GO:0003951 | NAD+ kinase activity | MOLECULAR_FUNCTION | 0.02 | 0.00 |
| GO:0036168 | filamentous growth of a population of unicellular organisms in response to heat | BIOLOGICAL_PROCESS | 0.02 | 0.00 |
| GO:0045947 | negative regulation of translational initiation | BIOLOGICAL_PROCESS | 0.02 | 0.00 |
| GO:1904688 | regulation of cytoplasmic translational initiation | BIOLOGICAL_PROCESS | 0.02 | 0.00 |
| GO:0070897 | transcription preinitiation complex assembly | BIOLOGICAL_PROCESS | 0.02 | 0.00 |
| GO:0008615 | pyridoxine biosynthetic process | BIOLOGICAL_PROCESS | 0.02 | 0.00 |
| GO:1903205 | regulation of hydrogen peroxide-induced cell death | BIOLOGICAL_PROCESS | 0.02 | 0.00 |
| GO:0051455 | monopolar spindle attachment to meiosis I kinetochore | BIOLOGICAL_PROCESS | 0.02 | 0.00 |
| GO:0019218 | regulation of steroid metabolic process | BIOLOGICAL_PROCESS | 0.02 | 0.00 |
| GO:0006098 | pentose-phosphate shunt | BIOLOGICAL_PROCESS | 0.02 | 0.00 |
| GO:0003849 | 3-deoxy-7-phosphoheptulonate synthase activity | MOLECULAR_FUNCTION | 0.02 | 0.00 |
| GO:0031400 | negative regulation of protein modification process | BIOLOGICAL_PROCESS | 0.02 | 0.00 |
| GO:0030120 | vesicle coat | CELLULAR_COMPONENT | 0.02 | 0.00 |
| GO:0140640 | catalytic activity, acting on a nucleic acid | MOLECULAR_FUNCTION | 0.02 | 0.00 |
| GO:2000142 | regulation of DNA-templated transcription, initiation | BIOLOGICAL_PROCESS | 0.02 | 0.00 |
| GO:0061645 | endocytic patch | CELLULAR_COMPONENT | 0.02 | 0.00 |
| GO:0030479 | actin cortical patch | CELLULAR_COMPONENT | 0.02 | 0.00 |
| GO:0099081 | supramolecular polymer | CELLULAR_COMPONENT | 0.02 | 0.00 |
| GO:0007267 | cell-cell signaling | BIOLOGICAL_PROCESS | 0.02 | 0.00 |
| GO:0042773 | ATP synthesis coupled electron transport | BIOLOGICAL_PROCESS | 0.02 | 0.00 |
| GO:0090502 | RNA phosphodiester bond hydrolysis, endonucleolytic | BIOLOGICAL_PROCESS | 0.02 | 0.00 |
| GO:0005730 | nucleolus | CELLULAR_COMPONENT | 0.02 | 0.00 |
| GO:0051287 | NAD binding | MOLECULAR_FUNCTION | 0.02 | 0.00 |
| GO:0009889 | regulation of biosynthetic process | BIOLOGICAL_PROCESS | 0.02 | 0.00 |
| GO:1902410 | mitotic cytokinetic process | BIOLOGICAL_PROCESS | 0.02 | 0.00 |
| GO:0045814 | negative regulation of gene expression, epigenetic | BIOLOGICAL_PROCESS | 0.02 | 0.00 |
| GO:0005759 | mitochondrial matrix | CELLULAR_COMPONENT | 0.02 | 0.00 |
| GO:0000422 | autophagy of mitochondrion | BIOLOGICAL_PROCESS | 0.02 | 0.00 |
| GO:0000460 | maturation of 5.8S rRNA | BIOLOGICAL_PROCESS | 0.02 | 0.00 |
| GO:0030880 | RNA polymerase complex | CELLULAR_COMPONENT | 0.02 | 0.00 |
| GO:0061726 | mitochondrion disassembly | BIOLOGICAL_PROCESS | 0.02 | 0.00 |
| GO:0006450 | regulation of translational fidelity | BIOLOGICAL_PROCESS | 0.02 | 0.00 |
| GO:0019897 | extrinsic component of plasma membrane | CELLULAR_COMPONENT | 0.02 | 0.00 |
| GO:0009250 | glucan biosynthetic process | BIOLOGICAL_PROCESS | 0.02 | 0.00 |
| GO:0005516 | calmodulin binding | MOLECULAR_FUNCTION | 0.02 | 0.00 |
| GO:0009890 | negative regulation of biosynthetic process | BIOLOGICAL_PROCESS | 0.02 | 0.00 |
| GO:0010556 | regulation of macromolecule biosynthetic process | BIOLOGICAL_PROCESS | 0.02 | 0.00 |
| GO:0006119 | oxidative phosphorylation | BIOLOGICAL_PROCESS | 0.02 | 0.00 |
| GO:0006720 | isoprenoid metabolic process | BIOLOGICAL_PROCESS | 0.02 | 0.00 |
| GO:0048534 | hematopoietic or lymphoid organ development | BIOLOGICAL_PROCESS | 0.02 | 0.00 |
| GO:0031428 | box C/D RNP complex | CELLULAR_COMPONENT | 0.02 | 0.00 |
| GO:0030097 | hemopoiesis | BIOLOGICAL_PROCESS | 0.02 | 0.00 |
| GO:0040029 | regulation of gene expression, epigenetic | BIOLOGICAL_PROCESS | 0.02 | 0.00 |
| GO:0009765 | photosynthesis, light harvesting | BIOLOGICAL_PROCESS | 0.02 | 0.00 |
| GO:0032527 | protein exit from endoplasmic reticulum | BIOLOGICAL_PROCESS | 0.02 | 0.00 |
| GO:0002520 | immune system development | BIOLOGICAL_PROCESS | 0.02 | 0.00 |
| GO:0006515 | protein quality control for misfolded or incompletely synthesized proteins | BIOLOGICAL_PROCESS | 0.02 | 0.00 |
| GO:0003954 | NADH dehydrogenase activity | MOLECULAR_FUNCTION | 0.02 | 0.00 |
| GO:1990351 | transporter complex | CELLULAR_COMPONENT | 0.02 | 0.00 |
| GO:0006534 | cysteine metabolic process | BIOLOGICAL_PROCESS | 0.02 | 0.00 |
| GO:0030687 | preribosome, large subunit precursor | CELLULAR_COMPONENT | 0.02 | 0.00 |
| GO:0046487 | glyoxylate metabolic process | BIOLOGICAL_PROCESS | 0.02 | 0.00 |
| GO:0043207 | response to external biotic stimulus | BIOLOGICAL_PROCESS | 0.02 | 0.00 |
| GO:0051707 | response to other organism | BIOLOGICAL_PROCESS | 0.02 | 0.00 |
| GO:0000118 | histone deacetylase complex | CELLULAR_COMPONENT | 0.02 | 0.00 |
| GO:0019902 | phosphatase binding | MOLECULAR_FUNCTION | 0.02 | 0.00 |
| GO:0006386 | termination of RNA polymerase III transcription | BIOLOGICAL_PROCESS | 0.02 | 0.00 |
| GO:0045041 | protein import into mitochondrial intermembrane space | BIOLOGICAL_PROCESS | 0.02 | 0.00 |
| GO:0035838 | growing cell tip | CELLULAR_COMPONENT | 0.02 | 0.00 |
| GO:0018193 | peptidyl-amino acid modification | BIOLOGICAL_PROCESS | 0.02 | 0.00 |
| GO:1902476 | chloride transmembrane transport | BIOLOGICAL_PROCESS | 0.02 | 0.00 |
| GO:0008540 | proteasome regulatory particle, base subcomplex | CELLULAR_COMPONENT | 0.02 | 0.00 |
| GO:0048308 | organelle inheritance | BIOLOGICAL_PROCESS | 0.02 | 0.00 |
| GO:0046847 | filopodium assembly | BIOLOGICAL_PROCESS | 0.02 | 0.00 |
| GO:0031625 | ubiquitin protein ligase binding | MOLECULAR_FUNCTION | 0.02 | 0.00 |
| GO:0050661 | NADP binding | MOLECULAR_FUNCTION | 0.02 | 0.00 |
| GO:0006081 | cellular aldehyde metabolic process | BIOLOGICAL_PROCESS | 0.02 | 0.00 |
| GO:0043038 | amino acid activation | BIOLOGICAL_PROCESS | 0.02 | 0.00 |
| GO:0070201 | regulation of establishment of protein localization | BIOLOGICAL_PROCESS | 0.02 | 0.00 |
| GO:0045214 | sarcomere organization | BIOLOGICAL_PROCESS | 0.02 | 0.00 |
| GO:0032968 | positive regulation of transcription elongation from RNA polymerase II promoter | BIOLOGICAL_PROCESS | 0.02 | 0.00 |
| GO:0006740 | NADPH regeneration | BIOLOGICAL_PROCESS | 0.02 | 0.00 |
| GO:0016779 | nucleotidyltransferase activity | MOLECULAR_FUNCTION | 0.02 | 0.00 |
| GO:0042221 | response to chemical | BIOLOGICAL_PROCESS | 0.02 | 0.00 |
| GO:0046686 | response to cadmium ion | BIOLOGICAL_PROCESS | 0.02 | 0.00 |
| GO:0016616 | oxidoreductase activity, acting on the CH-OH group of donors, NAD or NADP as acceptor | MOLECULAR_FUNCTION | 0.02 | 0.00 |
| GO:0046519 | sphingoid metabolic process | BIOLOGICAL_PROCESS | 0.02 | 0.00 |
| GO:0006030 | chitin metabolic process | BIOLOGICAL_PROCESS | 0.02 | 0.00 |
| GO:0000280 | nuclear division | BIOLOGICAL_PROCESS | 0.02 | 0.00 |
| GO:0051015 | actin filament binding | MOLECULAR_FUNCTION | 0.02 | 0.00 |
| GO:0044262 | cellular carbohydrate metabolic process | BIOLOGICAL_PROCESS | 0.02 | 0.00 |
| GO:0034703 | cation channel complex | CELLULAR_COMPONENT | 0.02 | 0.00 |
| GO:0031667 | response to nutrient levels | BIOLOGICAL_PROCESS | 0.02 | 0.00 |
| GO:0003746 | translation elongation factor activity | MOLECULAR_FUNCTION | 0.02 | 0.00 |
| GO:0004402 | histone acetyltransferase activity | MOLECULAR_FUNCTION | 0.02 | 0.00 |
| GO:0030218 | erythrocyte differentiation | BIOLOGICAL_PROCESS | 0.02 | 0.00 |
| GO:0034236 | protein kinase A catalytic subunit binding | MOLECULAR_FUNCTION | 0.02 | 0.00 |
| GO:1902743 | regulation of lamellipodium organization | BIOLOGICAL_PROCESS | 0.02 | 0.00 |
| GO:0034101 | erythrocyte homeostasis | BIOLOGICAL_PROCESS | 0.02 | 0.00 |
| GO:0002262 | myeloid cell homeostasis | BIOLOGICAL_PROCESS | 0.02 | 0.00 |
| GO:0033290 | eukaryotic 48S preinitiation complex | CELLULAR_COMPONENT | 0.02 | 0.00 |
| GO:0004674 | protein serine/threonine kinase activity | MOLECULAR_FUNCTION | 0.02 | 0.00 |
| GO:0034312 | diol biosynthetic process | BIOLOGICAL_PROCESS | 0.02 | 0.00 |
| GO:0034311 | diol metabolic process | BIOLOGICAL_PROCESS | 0.02 | 0.00 |
| GO:0072666 | establishment of protein localization to vacuole | BIOLOGICAL_PROCESS | 0.03 | 0.00 |
| GO:0044042 | glucan metabolic process | BIOLOGICAL_PROCESS | 0.03 | 0.00 |
| GO:0006073 | cellular glucan metabolic process | BIOLOGICAL_PROCESS | 0.03 | 0.00 |
| GO:0009991 | response to extracellular stimulus | BIOLOGICAL_PROCESS | 0.03 | 0.00 |
| GO:0022612 | gland morphogenesis | BIOLOGICAL_PROCESS | 0.03 | 0.00 |
| GO:1905905 | pharyngeal gland morphogenesis | BIOLOGICAL_PROCESS | 0.03 | 0.00 |
| GO:0098661 | inorganic anion transmembrane transport | BIOLOGICAL_PROCESS | 0.03 | 0.00 |
| GO:0072665 | protein localization to vacuole | BIOLOGICAL_PROCESS | 0.03 | 0.00 |
| GO:0010570 | regulation of filamentous growth | BIOLOGICAL_PROCESS | 0.03 | 0.00 |
| GO:0044389 | ubiquitin-like protein ligase binding | MOLECULAR_FUNCTION | 0.03 | 0.00 |
| GO:1902495 | transmembrane transporter complex | CELLULAR_COMPONENT | 0.03 | 0.00 |
| GO:0010557 | positive regulation of macromolecule biosynthetic process | BIOLOGICAL_PROCESS | 0.03 | 0.00 |
| GO:0000956 | nuclear-transcribed mRNA catabolic process | BIOLOGICAL_PROCESS | 0.03 | 0.00 |
| GO:0007154 | cell communication | BIOLOGICAL_PROCESS | 0.03 | 0.00 |
| GO:0006122 | mitochondrial electron transport, ubiquinol to cytochrome c | BIOLOGICAL_PROCESS | 0.03 | 0.00 |
| GO:0019954 | asexual reproduction | BIOLOGICAL_PROCESS | 0.03 | 0.00 |
| GO:0000220 | vacuolar proton-transporting V-type ATPase, V0 domain | CELLULAR_COMPONENT | 0.03 | 0.00 |
| GO:0006123 | mitochondrial electron transport, cytochrome c to oxygen | BIOLOGICAL_PROCESS | 0.03 | 0.00 |
| GO:0070069 | cytochrome complex | CELLULAR_COMPONENT | 0.03 | 0.00 |
| GO:0051156 | glucose 6-phosphate metabolic process | BIOLOGICAL_PROCESS | 0.03 | 0.00 |
| GO:0003727 | single-stranded RNA binding | MOLECULAR_FUNCTION | 0.03 | 0.00 |
| GO:0006418 | tRNA aminoacylation for protein translation | BIOLOGICAL_PROCESS | 0.03 | 0.00 |
| GO:0031382 | mating projection formation | BIOLOGICAL_PROCESS | 0.03 | 0.00 |
| GO:1990845 | adaptive thermogenesis | BIOLOGICAL_PROCESS | 0.03 | 0.00 |
| GO:1903513 | endoplasmic reticulum to cytosol transport | BIOLOGICAL_PROCESS | 0.03 | 0.00 |
| GO:0031521 | spitzenkorper | CELLULAR_COMPONENT | 0.03 | 0.00 |
| GO:0005199 | structural constituent of cell wall | MOLECULAR_FUNCTION | 0.03 | 0.00 |
| GO:1902953 | positive regulation of ER to Golgi vesicle-mediated transport | BIOLOGICAL_PROCESS | 0.03 | 0.00 |
| GO:0046354 | mannan biosynthetic process | BIOLOGICAL_PROCESS | 0.03 | 0.00 |
| GO:0072379 | ER membrane insertion complex | CELLULAR_COMPONENT | 0.03 | 0.00 |
| GO:0007105 | cytokinesis, site selection | BIOLOGICAL_PROCESS | 0.03 | 0.00 |
| GO:0044165 | host cell endoplasmic reticulum | CELLULAR_COMPONENT | 0.03 | 0.00 |
| GO:0032186 | cellular bud neck septin ring organization | BIOLOGICAL_PROCESS | 0.03 | 0.00 |
| GO:0030970 | retrograde protein transport, ER to cytosol | BIOLOGICAL_PROCESS | 0.03 | 0.00 |
| GO:0042973 | glucan endo-1,3-beta-D-glucosidase activity | MOLECULAR_FUNCTION | 0.03 | 0.00 |
| GO:0010412 | mannan metabolic process | BIOLOGICAL_PROCESS | 0.03 | 0.00 |
| GO:0009423 | chorismate biosynthetic process | BIOLOGICAL_PROCESS | 0.03 | 0.00 |
| GO:0010515 | negative regulation of induction of conjugation with cellular fusion | BIOLOGICAL_PROCESS | 0.03 | 0.00 |
| GO:0006357 | regulation of transcription by RNA polymerase II | BIOLOGICAL_PROCESS | 0.03 | 0.00 |
| GO:0008614 | pyridoxine metabolic process | BIOLOGICAL_PROCESS | 0.03 | 0.00 |
| GO:1903146 | regulation of autophagy of mitochondrion | BIOLOGICAL_PROCESS | 0.03 | 0.00 |
| GO:0006116 | NADH oxidation | BIOLOGICAL_PROCESS | 0.03 | 0.00 |
| GO:0033655 | host cell cytoplasm part | CELLULAR_COMPONENT | 0.03 | 0.00 |
| GO:0016875 | ligase activity, forming carbon-oxygen bonds | MOLECULAR_FUNCTION | 0.03 | 0.00 |
| GO:0004812 | aminoacyl-tRNA ligase activity | MOLECULAR_FUNCTION | 0.03 | 0.00 |
| GO:2000112 | regulation of cellular macromolecule biosynthetic process | BIOLOGICAL_PROCESS | 0.03 | 0.00 |
| GO:0001732 | formation of cytoplasmic translation initiation complex | BIOLOGICAL_PROCESS | 0.03 | 0.00 |
| GO:0005838 | proteasome regulatory particle | CELLULAR_COMPONENT | 0.03 | 0.00 |
| GO:0032786 | positive regulation of DNA-templated transcription, elongation | BIOLOGICAL_PROCESS | 0.03 | 0.00 |
| GO:0016114 | terpenoid biosynthetic process | BIOLOGICAL_PROCESS | 0.03 | 0.00 |
| GO:0031506 | cell wall glycoprotein biosynthetic process | BIOLOGICAL_PROCESS | 0.03 | 0.00 |
| GO:0042149 | cellular response to glucose starvation | BIOLOGICAL_PROCESS | 0.03 | 0.00 |
| GO:0048872 | homeostasis of number of cells | BIOLOGICAL_PROCESS | 0.03 | 0.00 |
| GO:0005826 | actomyosin contractile ring | CELLULAR_COMPONENT | 0.03 | 0.00 |
| GO:0009098 | leucine biosynthetic process | BIOLOGICAL_PROCESS | 0.03 | 0.00 |
| GO:0009051 | pentose-phosphate shunt, oxidative branch | BIOLOGICAL_PROCESS | 0.03 | 0.00 |
| GO:1990116 | ribosome-associated ubiquitin-dependent protein catabolic process | BIOLOGICAL_PROCESS | 0.03 | 0.00 |
| GO:0000032 | cell wall mannoprotein biosynthetic process | BIOLOGICAL_PROCESS | 0.03 | 0.00 |
| GO:0006057 | mannoprotein biosynthetic process | BIOLOGICAL_PROCESS | 0.03 | 0.00 |
| GO:0006056 | mannoprotein metabolic process | BIOLOGICAL_PROCESS | 0.03 | 0.00 |
| GO:0031010 | ISWI-type complex | CELLULAR_COMPONENT | 0.03 | 0.00 |
| GO:0030433 | ubiquitin-dependent ERAD pathway | BIOLOGICAL_PROCESS | 0.03 | 0.00 |
| GO:0030036 | actin cytoskeleton organization | BIOLOGICAL_PROCESS | 0.03 | 0.00 |
| GO:0051252 | regulation of RNA metabolic process | BIOLOGICAL_PROCESS | 0.03 | 0.00 |
| GO:0051094 | positive regulation of developmental process | BIOLOGICAL_PROCESS | 0.03 | 0.00 |
| GO:0060297 | regulation of sarcomere organization | BIOLOGICAL_PROCESS | 0.03 | 0.00 |
| GO:0060298 | positive regulation of sarcomere organization | BIOLOGICAL_PROCESS | 0.03 | 0.00 |
| GO:0016620 | oxidoreductase activity, acting on the aldehyde or oxo group of donors, NAD or NADP as acceptor | MOLECULAR_FUNCTION | 0.03 | 0.00 |
| GO:0051155 | positive regulation of striated muscle cell differentiation | BIOLOGICAL_PROCESS | 0.03 | 0.00 |
| GO:0004124 | cysteine synthase activity | MOLECULAR_FUNCTION | 0.03 | 0.00 |
| GO:0031126 | sno(s)RNA 3'-end processing | BIOLOGICAL_PROCESS | 0.03 | 0.00 |
| GO:0070993 | translation preinitiation complex | CELLULAR_COMPONENT | 0.03 | 0.00 |
| GO:0006873 | cellular ion homeostasis | BIOLOGICAL_PROCESS | 0.03 | 0.00 |
| GO:0006396 | RNA processing | BIOLOGICAL_PROCESS | 0.03 | 0.00 |
| GO:0006415 | translational termination | BIOLOGICAL_PROCESS | 0.03 | 0.00 |
| GO:0042775 | mitochondrial ATP synthesis coupled electron transport | BIOLOGICAL_PROCESS | 0.03 | 0.00 |
| GO:0051234 | establishment of localization | BIOLOGICAL_PROCESS | 0.03 | 0.00 |
| GO:0005938 | cell cortex | CELLULAR_COMPONENT | 0.03 | 0.00 |
| GO:0044769 | ATPase activity, coupled to transmembrane movement of ions, rotational mechanism | MOLECULAR_FUNCTION | 0.03 | 0.00 |
| GO:0042625 | ATPase-coupled ion transmembrane transporter activity | MOLECULAR_FUNCTION | 0.03 | 0.00 |
| GO:0046961 | proton-transporting ATPase activity, rotational mechanism | MOLECULAR_FUNCTION | 0.03 | 0.00 |
| GO:0031328 | positive regulation of cellular biosynthetic process | BIOLOGICAL_PROCESS | 0.03 | 0.00 |
| GO:0009891 | positive regulation of biosynthetic process | BIOLOGICAL_PROCESS | 0.03 | 0.00 |
| GO:0032880 | regulation of protein localization | BIOLOGICAL_PROCESS | 0.03 | 0.00 |
| GO:0019646 | aerobic electron transport chain | BIOLOGICAL_PROCESS | 0.03 | 0.00 |
| GO:0048193 | Golgi vesicle transport | BIOLOGICAL_PROCESS | 0.03 | 0.00 |
| GO:0006402 | mRNA catabolic process | BIOLOGICAL_PROCESS | 0.03 | 0.00 |
| GO:0000028 | ribosomal small subunit assembly | BIOLOGICAL_PROCESS | 0.03 | 0.00 |
| GO:0061733 | peptide-lysine-N-acetyltransferase activity | MOLECULAR_FUNCTION | 0.03 | 0.00 |
| GO:0040014 | regulation of multicellular organism growth | BIOLOGICAL_PROCESS | 0.03 | 0.00 |
| GO:0032956 | regulation of actin cytoskeleton organization | BIOLOGICAL_PROCESS | 0.03 | 0.00 |
| GO:0046503 | glycerolipid catabolic process | BIOLOGICAL_PROCESS | 0.03 | 0.00 |
| GO:0008406 | gonad development | BIOLOGICAL_PROCESS | 0.03 | 0.00 |
| GO:0045137 | development of primary sexual characteristics | BIOLOGICAL_PROCESS | 0.03 | 0.00 |
| GO:0030060 | L-malate dehydrogenase activity | MOLECULAR_FUNCTION | 0.03 | 0.00 |
| GO:0050017 | L-3-cyanoalanine synthase activity | MOLECULAR_FUNCTION | 0.03 | 0.00 |
| GO:0003968 | RNA-directed 5'-3' RNA polymerase activity | MOLECULAR_FUNCTION | 0.03 | 0.00 |
| GO:0070628 | proteasome binding | MOLECULAR_FUNCTION | 0.03 | 0.00 |
| GO:0005732 | sno(s)RNA-containing ribonucleoprotein complex | CELLULAR_COMPONENT | 0.03 | 0.00 |
| GO:0006810 | transport | BIOLOGICAL_PROCESS | 0.03 | 0.00 |
| GO:0098813 | nuclear chromosome segregation | BIOLOGICAL_PROCESS | 0.04 | 0.00 |
| GO:0045202 | synapse | CELLULAR_COMPONENT | 0.04 | 0.00 |
| GO:0003712 | transcription coregulator activity | MOLECULAR_FUNCTION | 0.04 | 0.00 |
| GO:0035384 | thioester biosynthetic process | BIOLOGICAL_PROCESS | 0.04 | 0.00 |
| GO:0071616 | acyl-CoA biosynthetic process | BIOLOGICAL_PROCESS | 0.04 | 0.00 |
| GO:0051703 | biological process involved in intraspecies interaction between organisms | BIOLOGICAL_PROCESS | 0.04 | 0.00 |
| GO:0006721 | terpenoid metabolic process | BIOLOGICAL_PROCESS | 0.04 | 0.00 |
| GO:0019888 | protein phosphatase regulator activity | MOLECULAR_FUNCTION | 0.04 | 0.00 |
| GO:0016020 | membrane | CELLULAR_COMPONENT | 0.04 | 0.00 |
| GO:0006730 | one-carbon metabolic process | BIOLOGICAL_PROCESS | 0.04 | 0.00 |
| GO:0000082 | G1/S transition of mitotic cell cycle | BIOLOGICAL_PROCESS | 0.04 | 0.00 |
| GO:0051153 | regulation of striated muscle cell differentiation | BIOLOGICAL_PROCESS | 0.04 | 0.00 |
| GO:0048732 | gland development | BIOLOGICAL_PROCESS | 0.04 | 0.00 |
| GO:0070382 | exocytic vesicle | CELLULAR_COMPONENT | 0.04 | 0.00 |
| GO:0006360 | transcription by RNA polymerase I | BIOLOGICAL_PROCESS | 0.04 | 0.00 |
| GO:0045211 | postsynaptic membrane | CELLULAR_COMPONENT | 0.04 | 0.00 |
| GO:0097730 | non-motile cilium | CELLULAR_COMPONENT | 0.04 | 0.00 |
| GO:0140513 | nuclear protein-containing complex | CELLULAR_COMPONENT | 0.04 | 0.00 |
| GO:0000775 | chromosome, centromeric region | CELLULAR_COMPONENT | 0.04 | 0.00 |
| GO:0010629 | negative regulation of gene expression | BIOLOGICAL_PROCESS | 0.04 | 0.00 |
| GO:0043235 | receptor complex | CELLULAR_COMPONENT | 0.04 | 0.00 |
| GO:0044877 | protein-containing complex binding | MOLECULAR_FUNCTION | 0.04 | 0.00 |
| GO:0007155 | cell adhesion | BIOLOGICAL_PROCESS | 0.04 | 0.00 |
| GO:0030150 | protein import into mitochondrial matrix | BIOLOGICAL_PROCESS | 0.04 | 0.00 |
| GO:0043039 | tRNA aminoacylation | BIOLOGICAL_PROCESS | 0.04 | 0.00 |
| GO:0019740 | nitrogen utilization | BIOLOGICAL_PROCESS | 0.04 | 0.00 |
| GO:0016469 | proton-transporting two-sector ATPase complex | CELLULAR_COMPONENT | 0.04 | 0.00 |
| GO:0040020 | regulation of meiotic nuclear division | BIOLOGICAL_PROCESS | 0.04 | 0.00 |
| GO:0034504 | protein localization to nucleus | BIOLOGICAL_PROCESS | 0.04 | 0.00 |
| GO:0098609 | cell-cell adhesion | BIOLOGICAL_PROCESS | 0.04 | 0.00 |
| GO:0035725 | sodium ion transmembrane transport | BIOLOGICAL_PROCESS | 0.04 | 0.00 |
| GO:0034243 | regulation of transcription elongation from RNA polymerase II promoter | BIOLOGICAL_PROCESS | 0.04 | 0.00 |
| GO:0030098 | lymphocyte differentiation | BIOLOGICAL_PROCESS | 0.04 | 0.00 |
| GO:0140658 | ATP-dependent chromatin remodeler activity | MOLECULAR_FUNCTION | 0.04 | 0.00 |
| GO:0005754 | mitochondrial proton-transporting ATP synthase, catalytic core | CELLULAR_COMPONENT | 0.04 | 0.00 |
| GO:0060628 | regulation of ER to Golgi vesicle-mediated transport | BIOLOGICAL_PROCESS | 0.04 | 0.00 |
| GO:0046084 | adenine biosynthetic process | BIOLOGICAL_PROCESS | 0.04 | 0.00 |
| GO:0046083 | adenine metabolic process | BIOLOGICAL_PROCESS | 0.04 | 0.00 |
| GO:0016241 | regulation of macroautophagy | BIOLOGICAL_PROCESS | 0.04 | 0.00 |
| GO:0061620 | glycolytic process through glucose-6-phosphate | BIOLOGICAL_PROCESS | 0.04 | 0.00 |
| GO:0061621 | canonical glycolysis | BIOLOGICAL_PROCESS | 0.04 | 0.00 |
| GO:0061718 | glucose catabolic process to pyruvate | BIOLOGICAL_PROCESS | 0.04 | 0.00 |
| GO:0046649 | lymphocyte activation | BIOLOGICAL_PROCESS | 0.04 | 0.00 |
| GO:0045321 | leukocyte activation | BIOLOGICAL_PROCESS | 0.04 | 0.00 |
| GO:0051179 | localization | BIOLOGICAL_PROCESS | 0.04 | 0.00 |
| GO:0002376 | immune system process | BIOLOGICAL_PROCESS | 0.04 | 0.00 |
| GO:0051300 | spindle pole body organization | BIOLOGICAL_PROCESS | 0.04 | 0.00 |
| GO:0005971 | ribonucleoside-diphosphate reductase complex | CELLULAR_COMPONENT | 0.04 | 0.00 |
| GO:0046417 | chorismate metabolic process | BIOLOGICAL_PROCESS | 0.04 | 0.00 |
| GO:0010520 | regulation of reciprocal meiotic recombination | BIOLOGICAL_PROCESS | 0.04 | 0.00 |
| GO:0045267 | proton-transporting ATP synthase, catalytic core | CELLULAR_COMPONENT | 0.04 | 0.00 |
| GO:0140453 | protein aggregate center | CELLULAR_COMPONENT | 0.04 | 0.00 |
| GO:0070863 | positive regulation of protein exit from endoplasmic reticulum | BIOLOGICAL_PROCESS | 0.04 | 0.00 |
| GO:0070861 | regulation of protein exit from endoplasmic reticulum | BIOLOGICAL_PROCESS | 0.04 | 0.00 |
| GO:0010971 | positive regulation of G2/M transition of mitotic cell cycle | BIOLOGICAL_PROCESS | 0.04 | 0.00 |
| GO:0033592 | RNA strand annealing activity | MOLECULAR_FUNCTION | 0.04 | 0.00 |
| GO:1903131 | mononuclear cell differentiation | BIOLOGICAL_PROCESS | 0.04 | 0.00 |
| GO:0033644 | host cell membrane | CELLULAR_COMPONENT | 0.04 | 0.00 |
| GO:0019343 | cysteine biosynthetic process via cystathionine | BIOLOGICAL_PROCESS | 0.04 | 0.00 |
| GO:0002521 | leukocyte differentiation | BIOLOGICAL_PROCESS | 0.04 | 0.00 |
| GO:0032970 | regulation of actin filament-based process | BIOLOGICAL_PROCESS | 0.04 | 0.00 |
| GO:0000428 | DNA-directed RNA polymerase complex | CELLULAR_COMPONENT | 0.04 | 0.00 |
| GO:0007264 | small GTPase mediated signal transduction | BIOLOGICAL_PROCESS | 0.04 | 0.00 |
| GO:0120032 | regulation of plasma membrane bounded cell projection assembly | BIOLOGICAL_PROCESS | 0.04 | 0.00 |
| GO:0060491 | regulation of cell projection assembly | BIOLOGICAL_PROCESS | 0.04 | 0.00 |
| GO:0043254 | regulation of protein-containing complex assembly | BIOLOGICAL_PROCESS | 0.04 | 0.00 |
| GO:0006739 | NADP metabolic process | BIOLOGICAL_PROCESS | 0.04 | 0.00 |
| GO:0098562 | cytoplasmic side of membrane | CELLULAR_COMPONENT | 0.04 | 0.00 |
| GO:0007600 | sensory perception | BIOLOGICAL_PROCESS | 0.04 | 0.00 |
| GO:0098754 | detoxification | BIOLOGICAL_PROCESS | 0.04 | 0.00 |
| GO:0006352 | DNA-templated transcription, initiation | BIOLOGICAL_PROCESS | 0.04 | 0.00 |
| GO:0030015 | CCR4-NOT core complex | CELLULAR_COMPONENT | 0.04 | 0.00 |
| GO:0006086 | acetyl-CoA biosynthetic process from pyruvate | BIOLOGICAL_PROCESS | 0.04 | 0.00 |
| GO:1902806 | regulation of cell cycle G1/S phase transition | BIOLOGICAL_PROCESS | 0.04 | 0.00 |
| GO:0019208 | phosphatase regulator activity | MOLECULAR_FUNCTION | 0.04 | 0.00 |
| GO:0046661 | male sex differentiation | BIOLOGICAL_PROCESS | 0.04 | 0.00 |
| GO:0036464 | cytoplasmic ribonucleoprotein granule | CELLULAR_COMPONENT | 0.05 | 0.00 |
| GO:0035770 | ribonucleoprotein granule | CELLULAR_COMPONENT | 0.05 | 0.00 |
| GO:0008610 | lipid biosynthetic process | BIOLOGICAL_PROCESS | 0.05 | 0.00 |
| GO:0001727 | lipid kinase activity | MOLECULAR_FUNCTION | 0.05 | 0.00 |
| GO:0097255 | R2TP complex | CELLULAR_COMPONENT | 0.05 | 0.00 |
| GO:0035095 | behavioral response to nicotine | BIOLOGICAL_PROCESS | 0.05 | 0.00 |
| GO:0048311 | mitochondrion distribution | BIOLOGICAL_PROCESS | 0.05 | 0.00 |
| GO:0009240 | isopentenyl diphosphate biosynthetic process | BIOLOGICAL_PROCESS | 0.05 | 0.00 |
| GO:0004535 | poly(A)-specific ribonuclease activity | MOLECULAR_FUNCTION | 0.05 | 0.00 |
| GO:0004568 | chitinase activity | MOLECULAR_FUNCTION | 0.05 | 0.00 |
| GO:0046490 | isopentenyl diphosphate metabolic process | BIOLOGICAL_PROCESS | 0.05 | 0.00 |
| GO:0006032 | chitin catabolic process | BIOLOGICAL_PROCESS | 0.05 | 0.00 |
| GO:0007059 | chromosome segregation | BIOLOGICAL_PROCESS | 0.05 | 0.00 |
| GO:0071688 | striated muscle myosin thick filament assembly | BIOLOGICAL_PROCESS | 0.05 | 0.00 |
| GO:0006376 | mRNA splice site selection | BIOLOGICAL_PROCESS | 0.05 | 0.00 |
| GO:0030029 | actin filament-based process | BIOLOGICAL_PROCESS | 0.05 | 0.00 |
| GO:0030684 | preribosome | CELLULAR_COMPONENT | 0.05 | 0.00 |
| GO:0030003 | cellular cation homeostasis | BIOLOGICAL_PROCESS | 0.05 | 0.00 |
| GO:0031349 | positive regulation of defense response | BIOLOGICAL_PROCESS | 0.05 | 0.00 |
| GO:0070783 | growth of unicellular organism as a thread of attached cells | BIOLOGICAL_PROCESS | 0.05 | 0.00 |
| GO:0019219 | regulation of nucleobase-containing compound metabolic process | BIOLOGICAL_PROCESS | 0.05 | 0.00 |
| GO:0006108 | malate metabolic process | BIOLOGICAL_PROCESS | 0.05 | 0.00 |
| GO:0006623 | protein targeting to vacuole | BIOLOGICAL_PROCESS | 0.05 | 0.00 |
| GO:0022624 | proteasome accessory complex | CELLULAR_COMPONENT | 0.05 | 0.00 |
| GO:0051149 | positive regulation of muscle cell differentiation | BIOLOGICAL_PROCESS | 0.05 | 0.00 |
| GO:0000154 | rRNA modification | BIOLOGICAL_PROCESS | 0.05 | 0.00 |
| GO:0048475 | coated membrane | CELLULAR_COMPONENT | 0.05 | 0.00 |
| GO:0006458 | 'de novo' protein folding | BIOLOGICAL_PROCESS | 0.05 | 0.00 |
| GO:0030117 | membrane coat | CELLULAR_COMPONENT | 0.05 | 0.00 |
| GO:0000097 | sulfur amino acid biosynthetic process | BIOLOGICAL_PROCESS | 0.05 | 0.00 |
| GO:1902562 | H4 histone acetyltransferase complex | CELLULAR_COMPONENT | 0.05 | 0.00 |
| GO:1905368 | peptidase complex | CELLULAR_COMPONENT | 0.05 | 0.00 |
| GO:0006979 | response to oxidative stress | BIOLOGICAL_PROCESS | 0.05 | 0.00 |
