# Supplementary material for: Deciphering the mechanism of anhydrobiosis in the entomopathogenic nematode Heterorhabditis indica through comparative transcriptomics
Source: PLoS One. 2022 Oct 27;17(10):e0275342. doi: 10.1371/journal.pone.0275342 (PMC9612587; doi:10.1371/journal.pone.0275342)
Supplement: Attachment — Submitted filename: Response to Reviewers.doc [file pone.0275342.s029.doc]

**Reviewer 1:**

| **Comment 1.**  The material and methods are well written, however what was the source of IJs of *Heterorhabditis* *indica* strain KX601067 and on what basis it was identified, is not mentioned. | The EPN *Heterorhabditis indica* was isolated from the IARI farm, New Delhi (Mohan *et al*., 2016). The details are included in the revised manuscript. |
| --- | --- |
| **Comment 2.**  Concrete conclusion about application of this research is lacking. Add one or two lines of conclusion in light of future application of this research. | As suggested the lines have been added at the end of the Discussion |

**Reviewer 2:**

| **Comment 1.**  In line 86, authors have mentioned that the read length was between 30 and 81 bp. Authors need to elaborate more on it. The information on whether it was single end or paired end sequencing is also not provided in the Materials and methods section. It is highly unlikely that read lengths were between 30-81 bp. Such a small length will give a misassembled de novo transcriptome. Detailed information on the sequencing depth, number of reads, purity filtered reads and elimination criteria for shortlisting the purity filtered reads should be provided in the M&M. | We have performed paired-end sequencing using Illumina Hi-Seq 2500 platform. The average sequence length of the trimmed reads was 81 bp. The details on the sequencing depth, number of reads, purity filtered reads and elimination criteria for shortlisting the purity filtered reads are included in the revised manuscript (Supplementary Table 1 a,b) |
| --- | --- |
| **Comment 2.**  Though authors have randomised the experiment, there is no information on the number of biological replicates used in the study. In case a single replicate was used, authors should compare at least their assembly from control samples with the previously published transcriptome data (Somvanshi et al., 2016). | Our analysis comprised of pooled samples from three independent biological replicates using 5000 IJs for each. Further, transcriptome library comparative assembly statistics using (*assembly stats* of *Trinity*) reveal that our transcriptome library statistics are relatively better than the transcriptome assembly of Somvanshi *et al*. (2016) by the following means: For example, N50 value of ours is 2843bp while it is 1291bp. Similarly, other parameters like N60, N70, N80, N90 and N100 are also higher in our library. Also, our sequence is clean as there are no ambiguous bases, whereas, in Somvanshi et al. (2016) it is 1670. Our assembly does not have any gaps, while it is 66 in number for them. So comparatively our assembly is superior in many ways compared to theirs. The details are included in Supplementary Table 6 in the manuscript. Parameter \| Our analysis \| Somvanshi et al., 2016; No. of reads \| 153050846 \| 20749541; No. of transcripts \| 93932 \| 23827; Avg. Transcript size \| 1629.38 \| 870.84; Largest transcript size \| 22658 \| 12876; N50 \| 2843 \| 1291; N60 \| 2355 \| 1036; N70 \| 1892 \| 787; N80 \| 1393 \| 578; N90 \| 764 \| 383; N100 \| 201 \| 100; N_count \| 0 \| 1670; Gaps \| 0 \| 66 We have performed BLASTn analysis between the two transcriptome and got hits around 700. Irrespective of changing parameters we could get hits between 600 to 700. In order cross verify we have performed alignment of (Raw reads from the database) transcriptome of Somvanshi *et al*., 2016 against the draft genome published by Bhat *et al*., 2022. The results are as follows: Bioinformatic tool \| Alignment rate of our data (Fresh, Anhydrobiotic) \| Alignment rate of Somvanshi et al., 2016; Bowtie2 \| 58.12%, 67.34% \| 0.05%; HISAT2 \| 73.79%, 90.29% \| 0.02% We have also mapped the transcriptome of Somvanshi *et al*., 2016 over the draft genome of Bhat *et al*., 2022. We have got similar results i.e. <1 % mapping. The comparative analysis is included in the results section of the revised manuscript. |
| **Comment 3.**  Since the draft genome of the *H.indica* has recently been published (Bhat et al., 2022), authors are encouraged to perform a reference based mapping and quantification to improve their data. Alternatively, they can also map the de novo assembled transcriptome on the genome and remove the false and chimeric assemblies to improve the overall quality. | The genome submitted by Bhat *et al*. (2022) is a draft one without any annotations. We have mapped our transcriptome by using the draft genome as reference through *GMAP* bioinformatics tool. In reference-based assembly, 78,454 transcripts were mapped to the draft genome. Also, we have assembled our raw data from both the conditions with Draft genome of Bhat *et al*. (2022) as reference genome using *HISAT2* bioinformatics tool. We have found that the 73.79% of reads from control sample assembled against the genome, while 90.29% of reads from anhydrobiotic nematodes sample assembled against it. These details are included in the revised manuscript. |
| **Comment 4.**  The results are too short, whereas the discussion is too long. Infact discussion has portions of results. It is advised that results can be merged. | As advised the Results and the Discussion have been modified |
| **Comment 5.**  Some of the sections in M&M can be merged, for e.g., 4.2.2.2 can be merged with 4.2.2.3 and 4.2.2.4. Similarly, 4.2.2.5 can be merged with 4.2.2.6 | As advised the sections have been merged accordingly |
| **Comment 6.**  The data should be submitted to a public database and requisite information should be provided in the manuscript. | The data has been submitted in NCBI database  BioProject ID: PRJNA789679  Bio sample No.: SAMN24146495  SRA IDs: SRR17284459, SRR17284460 |
