# Supplementary material for: Deciphering the mechanism of anhydrobiosis in the entomopathogenic nematode Heterorhabditis indica through comparative transcriptomics
Source: PLoS One. 2022 Oct 27;17(10):e0275342. doi: 10.1371/journal.pone.0275342 (PMC9612587; doi:10.1371/journal.pone.0275342)
Supplement: Attachment — Submitted filename: Response to reviewers.docx [file pone.0275342.s030.docx]

Reviewer 3

| **Comments** | **Response** |
| --- | --- |
| 1. In the experimental setup made by the authors, it is not clear if a ~10% glycerol solution could be inducing a dehydration response per se. Why not lowering the humidity of the chamber (below 60%) to induce a dehydration response? | The preconditioning of juveniles in ~10% glycerol solution (97% RH), over a period of 2 days, helps in a slow and gradual adaptation towards anhydrobiosis. The juveniles enter into desiccated state and after which they can survive in the almost complete absence of water and revive upon rehydration.  These nematodes are very sensitive to water loss and only gradual water loss, over a period of time, enable the morphological and physiological adjustments to help nematodes enter anhydrobiosis. At lower RH (below 90%) the water loss is rapid which does not allow nematode metabolism to regulate timely. Hence, nematode exposed to lower RH dry quickly and do not adapt to survive well.  “…*During entry into anhydrobiosis, a gradual water loss occurs over time, as water content falls from 75-80% in active nematodes to 2-5% in anhydrobiotic forms (Demeure & Freckman 1981). Survival is best if nematodes dry slowly; most species are killed if drying occurs too quickly (Barrett 1991; Demeure & Freckman 1981)*….”  Under natural field conditions, lack of rainfall and irrigation coupled with extended period of heat pulls the moisture away from the soil. The surface-soil starts to dry first, and the dryness extends gradually vertically into the A, B and O horizons. This allows *Heterorhabditis* and other anhydrobiotic nematodes, mostly prevalent in these horizons, enter into anhydrobiosis.  Erkut (2011) induced similar anhydrobiosis in *C. elegans* at 98% RH over an extended period of 4 days using NaOH *(Erkut, C., Penkov, S., Khesbak, H., Vorkel, D., Verbavatz, J. M., Fahmy, K., & Kurzchalia, T. V. (2011). Trehalose renders the dauer larva of Caenorhabditis elegans resistant to extreme desiccation. Current biology: CB, 21(15), 1331–1336. https://doi.org/10.1016/j.cub.2011.06.064)*  Both, *C. elegans* and *Heterorhabditis* are closely related nematodes belonging to Order Rhabditida. Our lab is successfully using the protocol for inducing anhydrobiosis at 97% RH in *Heterorhabditis* juveniles (Manimaran *et al*., 2012, 2015). The reference has been added in the manuscript. |
| 2. The description of GOs and its graphical representations are below the standard (Figures 4 and 5). What are the statisticals related to GO analysis (number of mapped transcripts, p-value, FDR)? A table containg the GO data and all statistical analysis is preferable instead of pizza graphics | As per the referee’s comment the modifications in the analysis have been incorporated and representation has been made in a tabulated form (Figure 6, Supplementary Table 9) |
| 3. The authors performed a clustering analysis of qPCR data (Figures 7 and 8)? If not, I strongly advise in order to better see the clusters of similar expressed genes. | The clustering analysis has been already done |
